# High Uric Acid Promotes Stem Leydig Cell Senescence by CCDC90B Mediates Mitochondrial Quality Control Imbalance

**DOI:** 10.1111/cpr.70237

**Published:** 2026-06-04

**Authors:** Jiayu Huang, Ao Wang, Xiangyu Li, Peng Huang, Kaixuan Zeng, Lu Sun, Moxuan Li, Yixiang Chen, Jiancheng Wang

**Affiliations:** ^1^ Scientific Research Center The Seventh Affiliated Hospital of Sun Yat‐Sen University Shenzhen Guangdong China; ^2^ Department of Urology The Sixth Affiliated Hospital, Sun Yat‐Sen University Guangzhou Guangdong China; ^3^ Biomedical Innovation Center The Sixth Affiliated Hospital, Sun Yat‐Sen University Guangzhou Guangdong China; ^4^ Department of Traditional Chinese Medicine The Seventh Affiliated Hospital of Sun Yat‐Sen University Shenzhen Guangdong China; ^5^ School of Medicine Sun Yat‐Sen University Shenzhen Guangdong China; ^6^ Center for Stem Cell Biology and Tissue Engineering Key Laboratory for Stem Cells and Tissue Engineering Ministry of Education Sun Yat‐Sen University Guangzhou Guangdong China

**Keywords:** AAV, CCDC90B, cellular senescence, hyperuricemia, mitochondrial quality control, stem Leydig cells, testosterone, uric acid

## Abstract

Hyperuricemia is a common metabolic disease and an important risk factor for low testosterone levels in men. The functional homeostasis of stem Leydig cells (SLCs) is crucial for maintaining testosterone levels. However, the potential molecular mechanism of how high uric acid (UA) levels affect SLC function remains to be elucidated. Here, we reveal that at the single‐cell RNA sequencing level, SLCs exhibit senescence under high UA conditions. Mechanistically, UA binds to CCDC90B, leading to its significant accumulation within cells. This exacerbates the influx of calcium ions into mitochondria, resulting in mitochondrial quality control (MQC) imbalance. In addition, at the level of organoids and transgenic mice, we observe SLC senescence is alleviated and considerable testosterone recovery after AAV8‐CCDC90B treatment. In summary, these results indicate that SLC senescence under high UA is regulated in a MQC‐dependent manner, with CCDC90B being a key regulatory target. Meanwhile, AAV‐mediated gene therapy may offer a promising therapeutic approach for patients with low testosterone levels.

## Graphical abstract

In hyperuricemia, uric acid inhibits CCDC90B ubiquitination degradation by binding to it. Excessive CCDC90B induces mitochondrial calcium overload, leading to mitochondrial quality control imbalance and ultimately resulting in SLC senescence and decreased testosterone levels.

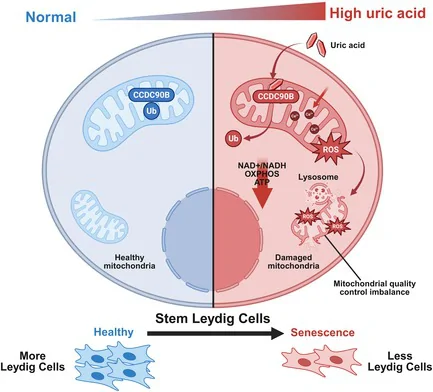

## 1 Introduction

Testosterone, primarily synthesised by Leydig cells (LCs), is essential for male reproductive development, spermatogenesis and systemic metabolic homeostasis [1, 2, 3]. The maintenance of testosterone production relies on stem Leydig cells (SLCs), which reside in the testicular interstitium and serve as progenitors for mature LCs [4, 5]. Through tightly regulated self‐renewal and differentiation, SLCs sustain LC populations and endocrine function under physiological conditions [6, 7]. Dysfunction of SLCs—manifested as impaired renewal, defective differentiation or premature senescence—directly leads to reduced testosterone levels and contributes to male hypogonadism and associated metabolic and reproductive disorders.

SLC differentiation into functional LCs is a multistep process tightly controlled by signals from the testicular microenvironment [8, 9]. This niche—comprising somatic cells, extracellular matrix components and soluble factors—coordinates the signalling networks that govern SLC fate decisions [10]. For instance, Hedgehog, Wnt and PI3K‐Akt pathways regulate SLC proliferation and differentiation and their disruption under pathological conditions impairs this process [9, 11, 12]. Given the sensitivity of the testicular microenvironment to metabolic disorders, understanding how a pathological metabolic state alters SLC function is critical for elucidating mechanisms of male reproductive dysfunction and developing therapeutic strategies.

In recent years, epidemiological evidence has shown that the increasing prevalence of metabolic diseases, such as obesity, insulin resistance and metabolic syndrome, is associated with reduced testosterone levels and impaired male fertility [13, 14, 15, 16]. Among these, hyperuricemia (defined as serum uric acid [UA] concentrations exceeding 7 mg/dL in men) represents a major component of metabolic abnormalities [17] and has shown a significant upward trend in adult males. Hyperuricemia has been extensively studied for its roles in oxidative stress, chronic inflammation and mitochondrial dysfunction, particularly, in tissues such as the kidney and joints [18, 19]. However, despite the high metabolic activity of the testis, the direct impact of elevated UA on SLC function remains largely unexplored.

Mitochondrial quality control (MQC) is essential for cellular energy metabolism and fate determination, encompassing mitochondrial biogenesis, dynamics (fusion and fission) and mitophagy [20, 21, 22, 23] Mitochondria dynamically respond to micro environmental stress and regulate cellular behaviour through MQC mechanisms [24, 25]. In stem cells, MQC homeostasis is critical for maintaining cellular function and its dysregulation induces senescence and impairs differentiation across multiple systems, including haematopoietic, nervous and skeletal tissues [26, 27]. Although MQC dysfunction in LCs has been linked to testosterone deficiency [28], its role in SLC differentiation and its disruption by elevated UA remain unclear.

In this study, we demonstrate that hyperuricemia reduces LC numbers and serum testosterone levels in mice. We hypothesised that SLC senescence underlies these alterations. Investigating the underlying causes of SLC senescence, we found that CCDC90B‐mediated MQC imbalance plays a pivotal role in driving UA‐induced SLC senescence. Specifically, excessive UA molecules bind to CCDC90B to enhance its resistance to proteasomal degradation, leading to intracellular CCDC90B accumulation, abnormally elevated mitochondrial calcium levels and subsequent MQC imbalance. Importantly, targeting CCDC90B using testicular organoid models and AAV‐based approaches alleviates SLC senescence and restores testosterone production. These findings reveal a previously unrecognised mechanism linking hyperuricemia to male reproductive dysfunction and provide potential therapeutic targets for related diseases.

## 2 Result

### 2.1 The Hyperuricemia Mice Show a Decrease in Testosterone Levels and Dysfunction of SLC Differentiation

To investigate the effect of hyperuricemia on systemic testosterone levels, we employed a Uox‐knockout mouse model on a pure C57BL/6J background (Uox^−/−^, Uox‐KO) as established in previous studies [29]. Testes and serum were harvested when the mice reached 8 weeks of age. We observed a significant decrease in testicular weight in Uox^−/−^ mice (Figure S1A,B). H&E staining revealed a significant reduction in the testicular interstitial area of Uox^−/−^ mice (Figure 1A,B). Concurrently, serum testosterone and testicular testosterone levels exhibited a marked decline (Figures 1C and S1C). Given that LCs are the primary cells for testosterone synthesis, qPCR analysis and immunofluorescence staining of LC‐related markers showed downregulated expression of CYP11A1, CYP17A1, HSD3β and STAR in the testes of Uox^−/−^ mice (Figures 1D and S1D–G). Subsequently, we quantified the number of LCs in the testes across different groups; flow cytometry results indicated a decrease in the LC population in Uox^−/−^ mice (Figure 1E,F). Meanwhile, LCs were found to be more susceptible to cell death under hyperuricemic conditions (Figures S1D and S1H). Furthermore, we cultured the sorted LCs to assess their testosterone‐synthesising capacity, which revealed no significant difference between LCs from WT and Uox^−/−^ mice (Figure 1G). Consequently, we hypothesised that SLC dysfunction might lead to an insufficient replenishment of the LC pool, thereby resulting in low testosterone levels. We obtained SLC from the testes of WT and Uox^−/−^ mice through flow cytometry based on the SLC specific marker CD51 [30, 31]. And examined the expression of LC‐related markers after induced differentiation; the results showed significantly lower expression in the Uox^−/−^ group (Figure 1H–K). These findings suggest that the low testosterone levels induced by hyperuricemia are likely associated with SLC dysfunction.

**FIGURE 1:**
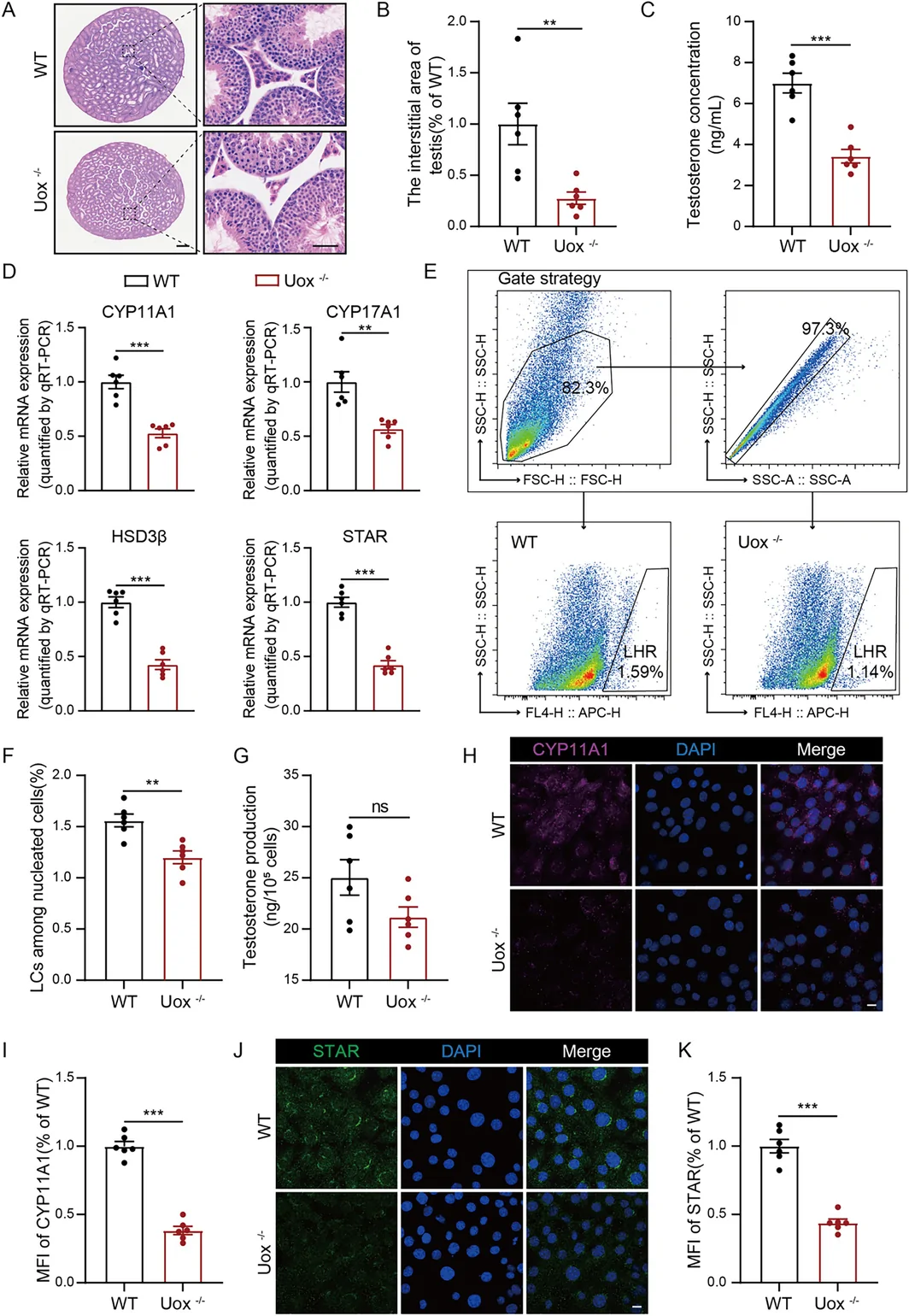
The hyperuricemia mice show a decrease in testosterone levels and dysfunction of SLC differentiation. (A) Representative H&E staining pictures of testicular mesenchyme from WT and Uox^−/−^ groups (*n* = 6 biological repeats for each group; left: Bar = 500 μm, right: Bar = 50 μm). (B) Quantification of mean area of the testicular interstitium in (A). (C) Measurement of testosterone concentration in the serum. (D) qPCR analysis of relative mRNA expression of LCs markers of testes from WT and Uox^−/−^ groups. (E) Flow cytometry for detecting the percentage of LHR^+^cells (luteinizing hormone receptor^+^cell) in the testis of WT and Uox^−/−^ groups. (F) Quantification of percentage of LHR^+^ cells in the testis of WT and Uox^−/−^ groups in (E). (G) Measurement of testosterone concentration in the medium after induced differentiation. (H) Representative immunofluorescence images of CYP11A1 after SLCs induced differentiation from WT and Uox^−/−^ groups (bar = 10 μm). (I) Quantification of mean fluorescence intensity (MFI) of CYP11A1 in (H). (J) Representative immunofluorescence images of STAR after SLCs induced differentiation from WT and Uox^−/−^ groups (bar = 10 μm). (K) Quantification of mean fluorescence intensity of STAR in (J). Data are represented as mean ± SEM. In all bar graphs, each dot represents one biological replicate. *p < 0.05, **p < 0.01, ***p < 0.001; ns, no significance by unpaired Student's *t*‐test (B‐D, F, G, I and K).

### 2.2 SLC Senescence Is Observed in the Testes of Mice With Hyperuricemia

To determine the intrinsic causes of SLC dysfunction under a hyperuricemic microenvironment, we utilised single‐cell RNA sequencing (scRNA‐seq) data (The number of cells is 67,697, the samples consist of three WT mouse testes and three Uox^−^/^−^ mouse testes) to investigate the pathophysiological changes in SLCs within hyperuricemic testes. Initially, we performed clustering analysis on all cell populations (Figures 2A and S2A). By observing the proportions of each cell cluster, we found that the ratios of LCs and SLCs were decreased in the testes of Uox^−/−^ mice (Figure 2B). Subsequently, we conducted functional enrichment analysis on SLCs to clarify the functional alterations occurring in the hyperuricemic testicular interstitial microenvironment. GO and GSEA results revealed an enrichment of senescence‐related genes in SLCs (Figures 2C,D and S2B). Furthermore, alterations in the typical gene expression profiles of SLCs showed higher expression of senescence‐associated genes in the Uox^−/−^ group (Figure S2C). Concurrently, SLCs from Uox^−/−^ mice exhibited higher senescence scores (Figure 2E). To further validate the scRNA‐seq findings, we isolated and cultured SLCs from mouse testes; the results showed significantly more SA‐β‐gal‐positive cells in the Uox^−/−^ group (Figure 2F,G). We examined the expression of senescence‐associated proteins in the testicular interstitium, which revealed a significant increase in p16^+^SLCs and p21^+^SLCs in Uox^−/−^ mice (Figure 2H–K). Meanwhile, the expression of senescence‐associated secretory phenotype (SASP) factors (IL‐1α, IL‐6, CXCL15, MMP‐14) was significantly elevated in the SLCs of Uox^−/−^ mice (Figure S2D). Additionally, previous studies have shown that UA can cause inflammatory reactions in tissues [32, 33]. We found that the levels of ROS and inflammatory factors (IL‐6, IL‐1β and TNF‐α) in the testes of Uox^−/−^ mice were significantly increased (Figure S2E–G). Therefore, we believe that tissue inflammation may exacerbate the effects of UA on SLCs and LCs. Collectively, these results demonstrate that SLC senescence in hyperuricemic mice is a critical factor leading to low testosterone levels.

**FIGURE 2:**
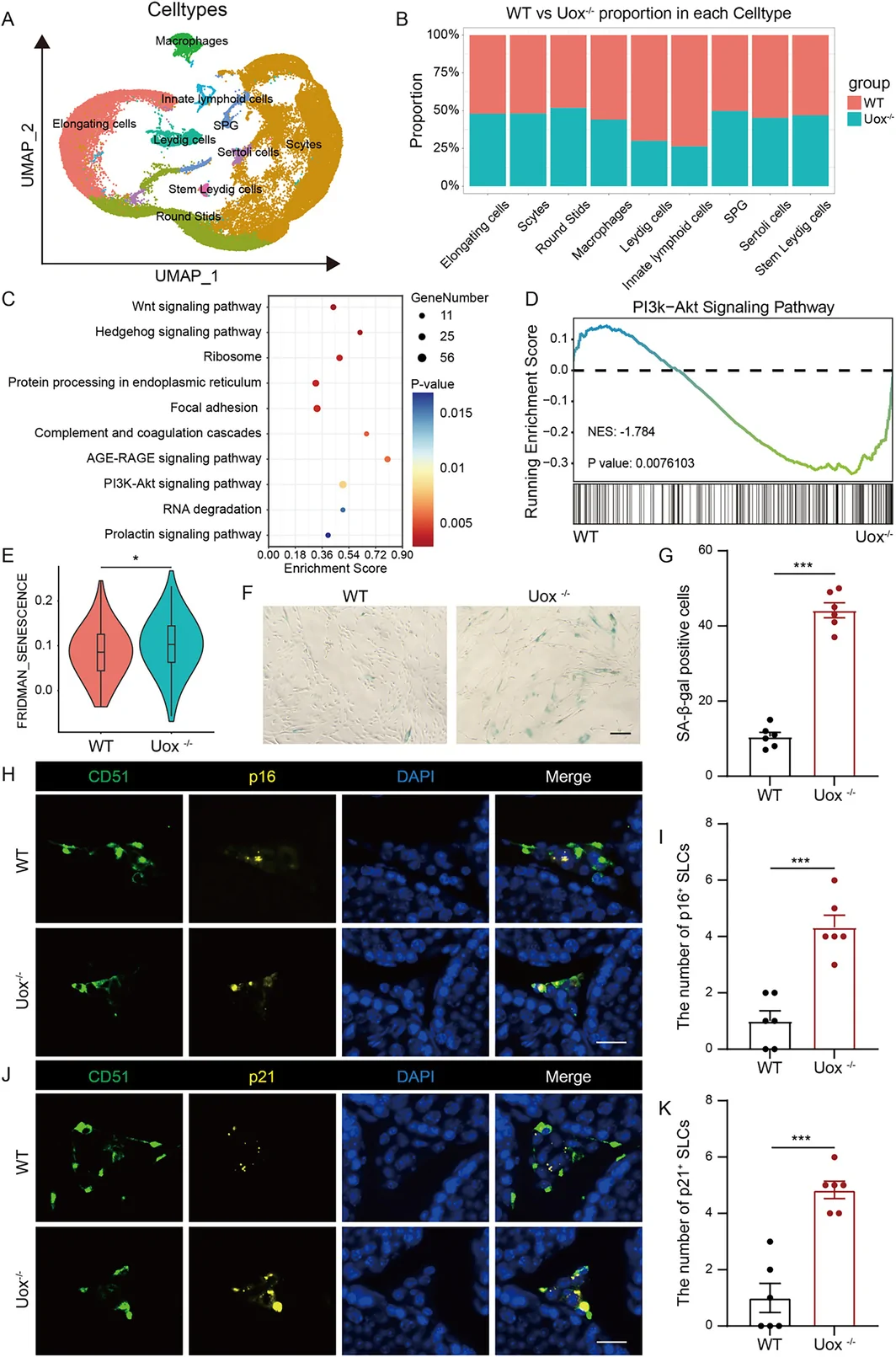
SLC senescence is observed in the testes of mice with hyperuricemia. (A) Cell clusters of the sc‐RNA‐seq data. (B) The histogram showed the proportions of various cells in WT and Uox^−/−^ mice samples. (C) The bubble plot showed that differentially expressed genes of SLCs in the WT group and Uox^−/−^ group were significantly enriched in the gene sets. (D) GSEA of the hallmark gene sets in the MSigDB database revealing the enrichment of PI3k‐Akt Signalling Pathway GO terms in SLCs. NES, normalised enrichment score. (E) The senescence scores of SLCs in WT and Uox^−/−^ groups. (F and G) SA‐β‐Gal images and quantification analysis showed the number of SA‐β‐Gal‐positive cells in SLCs. (H) Representative immunofluorescence images of p16^+^SLCs in testis paraffin sections from WT and Uox^−/−^ groups. (I) Quantification of the number of p16^+^SLCs in (H). (J) Representative immunofluorescence images of p21^+^SLCs in testis frozen sections from WT and Uox^−/−^ groups. (K) Quantification of the number of p21^+^SLCs in (J). Data are represented as mean ± SEM. In all bar graphs, each dot represents one biological replicate. *p < 0.05, **p < 0.01, ***p < 0.001; ns, no significance by unpaired Student's *t*‐test (G, I and K).

### 2.3 High UA Environment Promotes SLC Senescence and Inhibits Its Function

In order to obtain a large amount of SLCs for in vitro experiments in a simple and fast manner. We obtained WT mice testes, digested and filtered to obtain mixed cells and cultured them in SLC proliferation medium for three generations. The expression of SLC markers CD51 and Nestin [34, 35] was detected by flow cytometry. The results showed that after three generations of amplification, over 90% were SLCs (Figure S3A). Whereafter, a hyperuricemic environment was established by adding UA to the culture medium. Initially, we obtained a UA *IC50* fitting curve using the CCK‐8 assay and determined the working UA concentration to be 2.944 mM (Figure S3B). To detect dose dependence, we set different UA concentrations. It was found that as the concentration of UA increased, the ability of SLC to differentiate into LC and produce testosterone gradually decreased (Figure S3C). Subsequent experiments will fix the UA concentration at 2.944 mM. Cellular senescence was assessed after 48 h of UA exposure; the results showed that the hyperuricemic environment significantly increased the proportion of SA‐β‐gal positive cells (Figure 3A,B). Western blot analysis revealed that, compared to the control group, the levels of cell cycle‐related proteins p16, p21 and p53 were significantly elevated, while p‐RB levels were markedly reduced in the UA group (Figure 3C,D). Subsequently, we evaluated oxidative stress levels in SLCs and found a significant increase in ROS in the UA group (Figure 3E,F). Additionally, the expression levels of SASP factors in SLCs cultured under hyperuricemic conditions were significantly upregulated (Figure S3D–G). We performed functional assays on the SLCs. The clonal sphere formation assay demonstrated that SLCs cultured in the hyperuricemic environment exhibited weaker proliferation capacity than the control group (Figure 3G–I). Moreover, qPCR, immunofluorescence staining and Western blot analyses showed that the expression levels of LC‐related markers in SLCs following induced differentiation were significantly lower in the UA group compared to the control group (Figures 3J–L and S3H–K). Simultaneously, the detection of the cAMP/PKA pathway related to steroid synthesis revealed that high UA reduced the activation level of the cAMP/PKA signalling pathway (Figures 3M,N and S3L). These findings further demonstrate that high UA leads to low testosterone levels by inducing SLC senescence.

**FIGURE 3:**
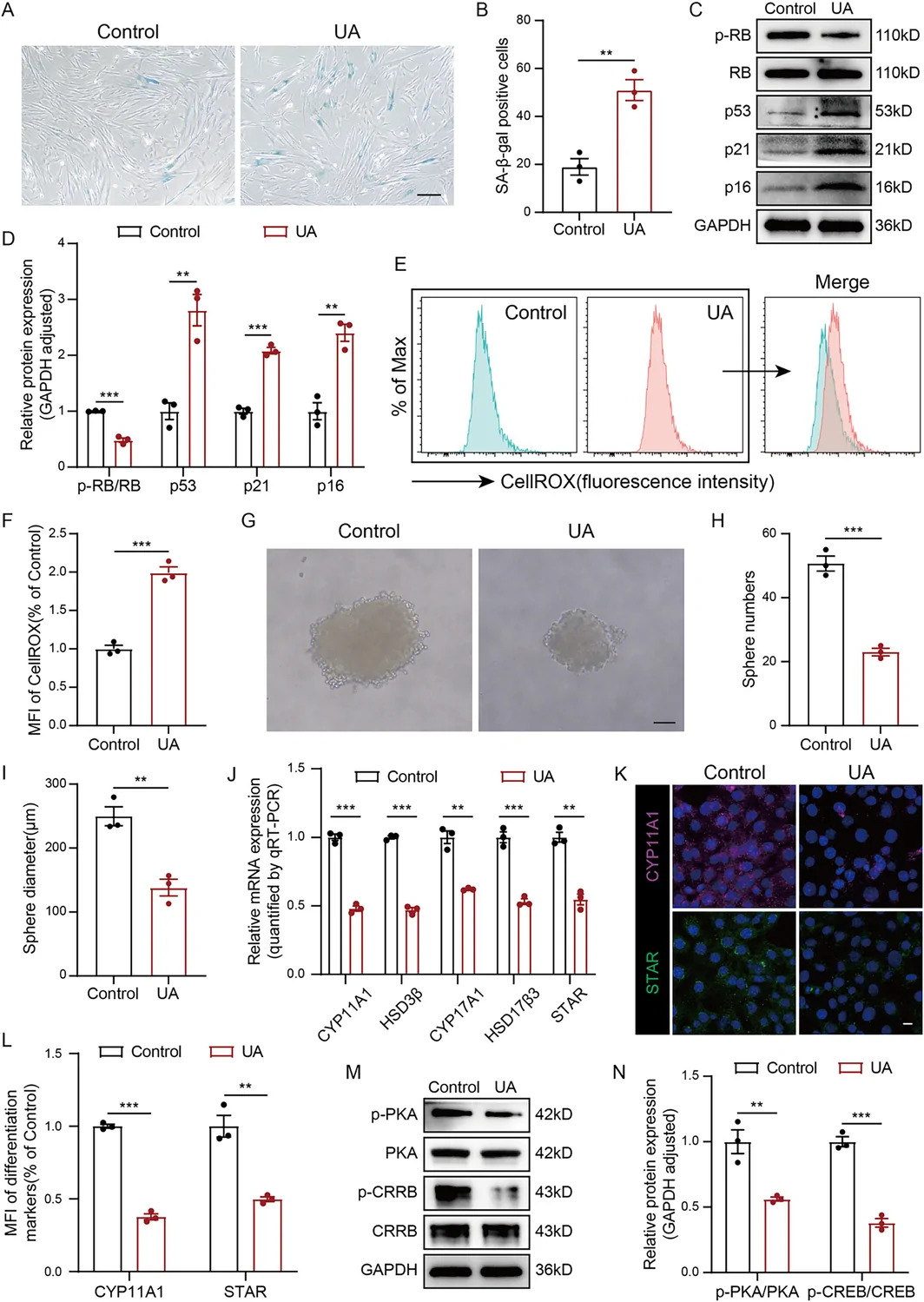
High uric acid environment promotes SLC senescence and inhibits its function. (A, B) SA‐β‐Gal images and quantification analysis showed the number of SA‐β‐Gal‐positive cells in SLCs. (C, D) Western Blot analysis and quantification of senescence marker expression in SLCs of Control and UA groups. (E) Flow cytometry of cell ROS levels stained with CellROX of SLCs of Control and UA groups. (F) Quantification of mean fluorescence intensity of CellROX in (E). (G) Spheres of representative images of the indicated SLCs from Control and UA groups. (bar = 50 μm). (H, I) Histograms show the mean numbers and diameters of spheres cultured. (J) qPCR analysis of relative mRNA expression of LC markers after SLCs induced differentiation of Control and UA groups. (K) Representative immunofluorescence images of LC markers after SLCs induced differentiation of Control and UA groups. (bar = 10 μm). (L) Quantification of mean fluorescence intensity of LC markers in (K). (M, N) Western Blot analysis and quantification of activation level of cAMP/PKA pathway after SLCs induced differentiation of Control and UA groups. Data are represented as mean ± SEM. In all bar graphs, each dot represents one biological replicate. *p < 0.05, **p < 0.01, ***p < 0.001; ns, no significance by unpaired Student's *t*‐test (B, D, F, H–J, L and N).

### 2.4 CCDC90B Mediates SLC Mitochondrial Quality Control Imbalance at High UA Environment

To further explore the molecular mechanisms underlying UA‐induced SLC senescence, we employed drug affinity responsive target stability (DARTS) technology coupled with proteomic analysis to screen and identify potential target proteins of UA, leveraging its small‐molecule properties (Figure 4A). Protein heatmap results revealed that CCDC90B was the most significantly differentially expressed protein (Figure 4B). In silico docking studies revealed that UA binds to the druggable pocket of CCDC90B (Figure 4C). Western blot analysis showed that CCDC90B protein levels were significantly higher in the UA group compared to the control group (Figure 4D,E). These results suggest that the binding of UA to CCDC90B reduces the susceptibility of CCDC90B to proteolytic degradation. Furthermore, we performed Co‐IP and Western blotting to examine the effect of UA on CCDC90B ubiquitination in SLCs; the results indicated a reduction in the ubiquitin‐mediated degradation of CCDC90B under hyperuricemic conditions (Figure S4A,B). Subsequently, we investigated the functional changes in SLCs resulting from elevated CCDC90B levels. CCDC90B is localised to the mitochondrial inner membrane and is a vital component of the mitochondrial calcium uniporter complex, playing a key role in regulating mitochondrial calcium uptake [36]. Therefore, we utilised the RHOD‐2 AM dye to measure mitochondrial calcium content in SLCs, finding that it was significantly elevated under hyperuricemic conditions (Figure 4F,G). Given that mitochondrial calcium homeostasis is crucial for maintaining mitochondrial function [37, 38], we hypothesised that high UA may impact SLC mitochondrial function via CCDC90B.

**FIGURE 4:**
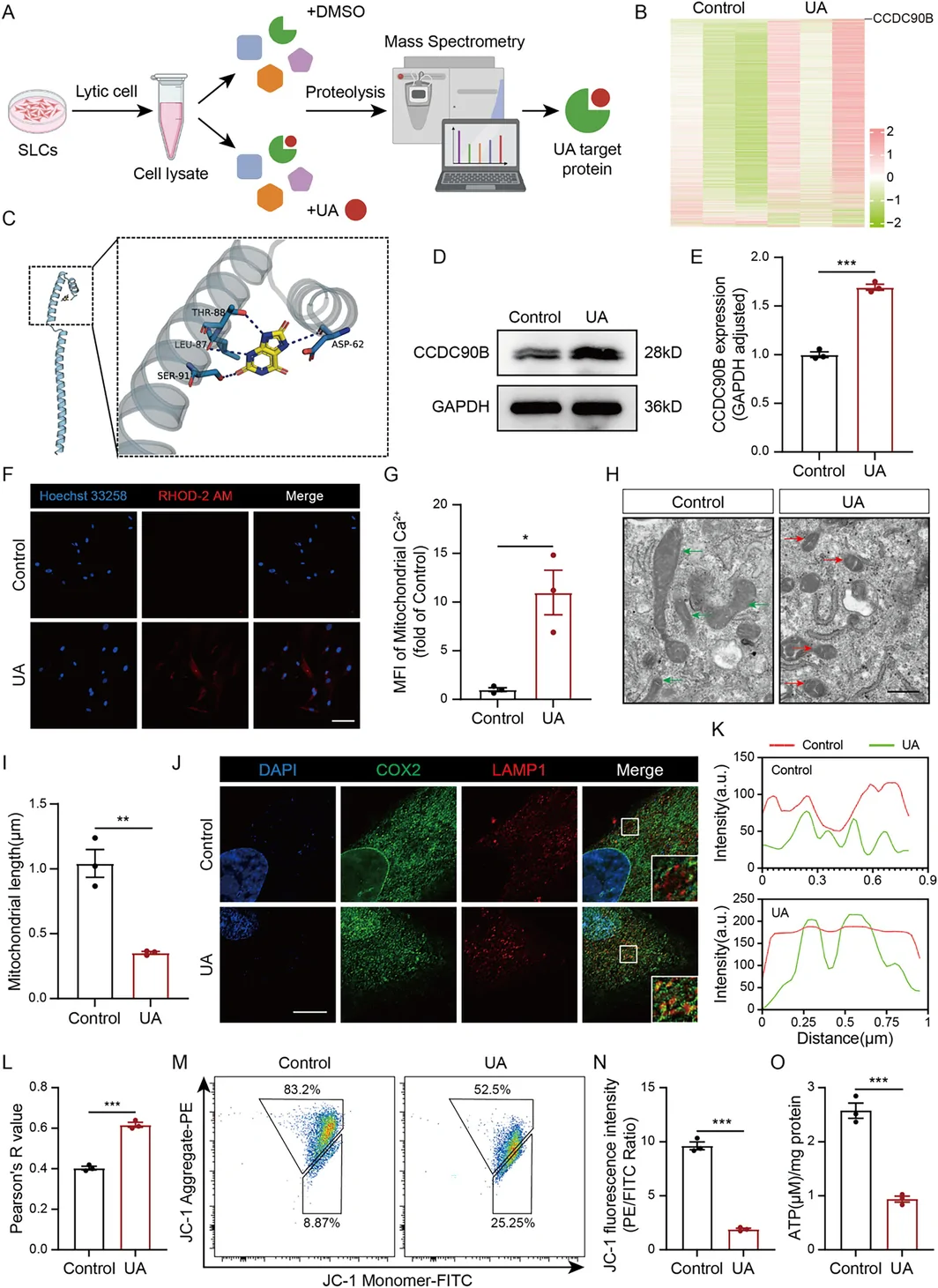
CCDC90B mediates SLC mitochondrial quality control imbalance at high uric acid environment. (A) Schematic diagram of DARTS assay. (B) The heat map showed the differential proteins in Control and UA groups. (C) The stereo view of MD‐optimised complex structure of CCDC90B bound with uric acid and the detailed interactions between CCDC90B and uric acid. (D, E) Western Blot analysis and quantification of CCDC90B expression in SLCs of Control and UA groups. (F) Representative immunofluorescence images of SLC mitochondrial calcium ion (RHOD‐2 AM, red+) of Control and UA groups (bar = 100 μm). (G) Quantification of mean fluorescence intensity of mitochondrial calcium ion in (F). (H) Representative transmission electron microscope images of the morphology of mitochondria in SLCs of Control and UA groups. Red and green arrows indicate damaged and normal mitochondria, respectively (bar = 500 nm). (I) Quantitative analysis of average value of mitochondrial length (μm) in (H). (J) Representative immunofluorescence images of COX2 and LAMP1 in SLCs of Control and UA groups (bar = 10 μm). (K) Colocalisation analysis of the fluorescence intensity in (J). (L) Quantitative statistical graph of (K). (M) Flow cytometry analysis of mitochondrial membrane potential (MMP) probed with JC‐1 in SLCs after treatment with uric acid for 24 h. (N) Quantitative analysis of the ratio of JC‐1 aggregates (referred to high MMP, PE channel)/JC‐1 monomers (referred to low MMP, FITC channel) in (M). (O) Measurement of the intracellular ATP level in SLCs of Control and UA groups. Data are represented as mean ± SEM. In all bar graphs, each dot represents one biological replicate. *p < 0.05, **p < 0.01, ***p < 0.001; ns, no significance by unpaired Student's t test (E, G, I, L, N and O).

Mitochondria are central to cellular survival and redox homeostasis and their dysfunction is closely linked to ageing and cell senescence [39, 40, 41, 42]. As SLC differentiation into LCs relies heavily on mitochondrial respiration, mitochondrial homeostasis is critical for maintaining SLC function. MQC plays a key role in preserving mitochondrial integrity and metabolic capacity [43]. Therefore, we further investigated the changes in MQC within ageing SLCs. We observed that SLCs cultured in a hyperuricemic environment exhibited a fragmented mitochondrial network morphology (Figures 4H,I and S4C,D). The results indicate a significant change in mitochondrial autophagy levels. Subsequently, we assessed mitophagy and found that its levels were higher in the UA group compared to the control (Figure 4J–L). Regarding mitochondrial oxidative stress, the UA‐treated SLCs exhibited a significant increase in mitochondrial ROS, along with decreased expression of intracellular redox proteins (Figure S4E–G). Regarding mitochondrial energy metabolism. High UA significantly inhibited SLC mitochondrial membrane potential and reduced mitochondrial ATP production, further indicating the disruption of mitochondrial homeostasis (Figure 4M–O). These findings demonstrate that MQC imbalance, mediated by the mitochondrial inner membrane protein CCDC90B, may be involved in influencing SLC senescence under hyperuricemic conditions.

### 2.5 Interference With CCDC90B Alleviates SLC Senescence Under High UA Conditions and Restore Its Differentiation Function

To further validate these findings, we utilised shRNA to knock down CCDC90B expression (hereafter abbreviated as shC). Results demonstrated that silencing CCDC90B effectively attenuated UA‐induced cellular senescence in SLCs. This was evidenced by a reduction in SA‐β‐gal‐positive cells (Figure 5A,B). At the protein aspect, interference with CCDC90B effectively reduced the protein levels of p53, p21 and p16, and increased the expression level of p‐RB (Figure 5C,D). At the mRNA level, interference with CCDC90B inhibited the expression of SASP factor (Figure 5E–H). Regarding MQC, CCDC90B knockdown lowered mitochondrial calcium content in UA‐treated SLCs and ameliorated mitochondrial fragmentation (Figure S5A–D). Concurrently, CCDC90B knockdown reduced mitochondrial ROS, enhanced mitochondrial membrane potential and ATP production and restored the expression of intracellular redox proteins, thereby recovering mitochondrial function (Figure S5E–J). In terms of SLC functionality, CCDC90B interference significantly restored the proliferation capacity of SLCs under hyperuricemic conditions (Figure 5I–K). Meanwhile, assays for LC‐related markers indicated that CCDC90B knockdown significantly rescued the differentiation potential of SLCs into LCs under high UA (Figure 5L–N). In the steroid synthesis‐related pathways, silencing CCDC90B can increase the activation level of the cAMP/PKA pathway in SLCs under high UA conditions (Figures 5O and S5K,L), consequently increasing testosterone levels (Figure 5P). In summary, our results indicate that high UA‐induced SLC senescence occurs in an MQC‐dependent manner, with CCDC90B playing a pivotal role in regulating this process.

**FIGURE 5:**
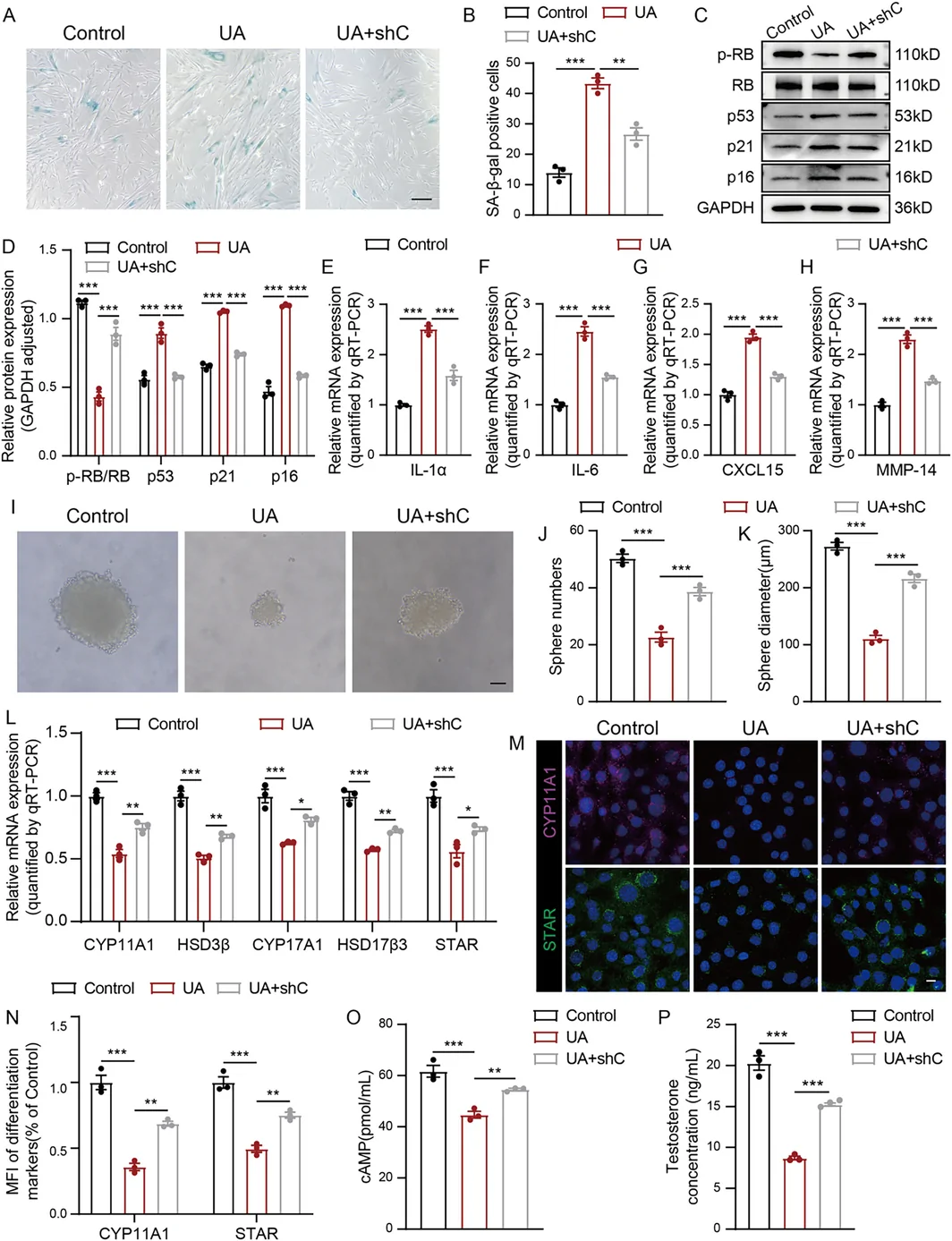
Interference with CCDC90B alleviates SLC senescence under high uric acid conditions and restores its differentiation function. (A, B) SA‐β‐Gal images and quantification analysis showed the number of SA‐β‐Gal‐positive cells in SLCs. (C, D) Western Blot analysis and quantification of senescence markers' expression in SLCs of Control, UA and UA + shCCDC90B (shC) groups. (E–H) qPCR analysis of relative mRNA expression of the SASP genes in SLCs of Control, UA and UA + shC groups. (I) Spheres of representative images of the indicated SLCs from Control, UA and UA + shC groups (bar = 50 μm). (J, K) Histograms show the mean numbers and diameters of spheres cultured. (L) qPCR analysis of relative mRNA expression of LC markers after SLCs induced differentiation of Control, UA and UA + shC groups. (M) Representative immunofluorescence images of LC markers after SLCs induced differentiation of Control, UA and UA + shC groups. (bar = 10 μm). (N) Quantification of mean fluorescence intensity in (M). (O) ELISA experiment for detecting cAMP levels after SLCs induced differentiation of Control, UA and UA + shC groups. (P) Measurement of testosterone concentration in the medium after induced differentiation. Data are represented as mean ± SEM. In all bar graphs, each dot represents one biological replicate. *p < 0.05, **p < 0.01, ***p < 0.001; ns, no significance by one‐way ANOVA (B, D–H, J–L and N–P).

### 2.6 Targeting CCDC90B Rescues Testicular Organoids From High UA‐Induced Inhibition via Mitochondrial Quality Control

Organoids, as three‐dimensional in vitro micro‐organs, possess genetic backgrounds and histological characteristics highly similar to those in vivo; their complex, organ‐like structures enable partial recapitulation of the source tissue, making them ideal research models [44]. Here, we constructed testicular organoids following the methodology reported by Alves‐Lopes et al. [45](Figure S6A). Subsequently, UA was added to the testicular organoid culture system to establish a hyperuricemic environment. Furthermore, based on our previously reported study [46], we utilised AAV8 to target and knockdown CCDC90B expression in the SLCs within the testicular organoids (Figure 6A). After 21 days of culture, we observed that the testicular organoids exhibited retarded growth under hyperuricemic conditions, the seminiferous tubule‐like structures in the organoids are not obvious (Figures 6B,C and S6B). Fluorescence imaging shows that AAV8 can specifically target SLCs and LCs in testicular organoids (Figure S6C). Regarding mitochondrial function, compared with the control group, the hyperuricemic group showed elevated mitochondrial calcium levels in the SLCs within the testicular organoids, whereas AAV8‐mediated CCDC90B interference attenuated these effects (Figure 6D). On the other hand, we evaluated senescence and functional indicators in the testicular organoids. Western blot analysis revealed that AAV8‐targeted CCDC90B interference reduced the protein levels of p16, p21 and p53, while increasing p‐RB levels in the hyperuricemic organoids (Figure S6D–E). qPCR results indicated that the expression of SASP factors was inhibited and the expression of LC‐related markers was upregulated (Figures 6E–G and S6F). Concurrently, testosterone levels in the testicular organoid culture medium were significantly increased (Figure 6H).

**FIGURE 6:**
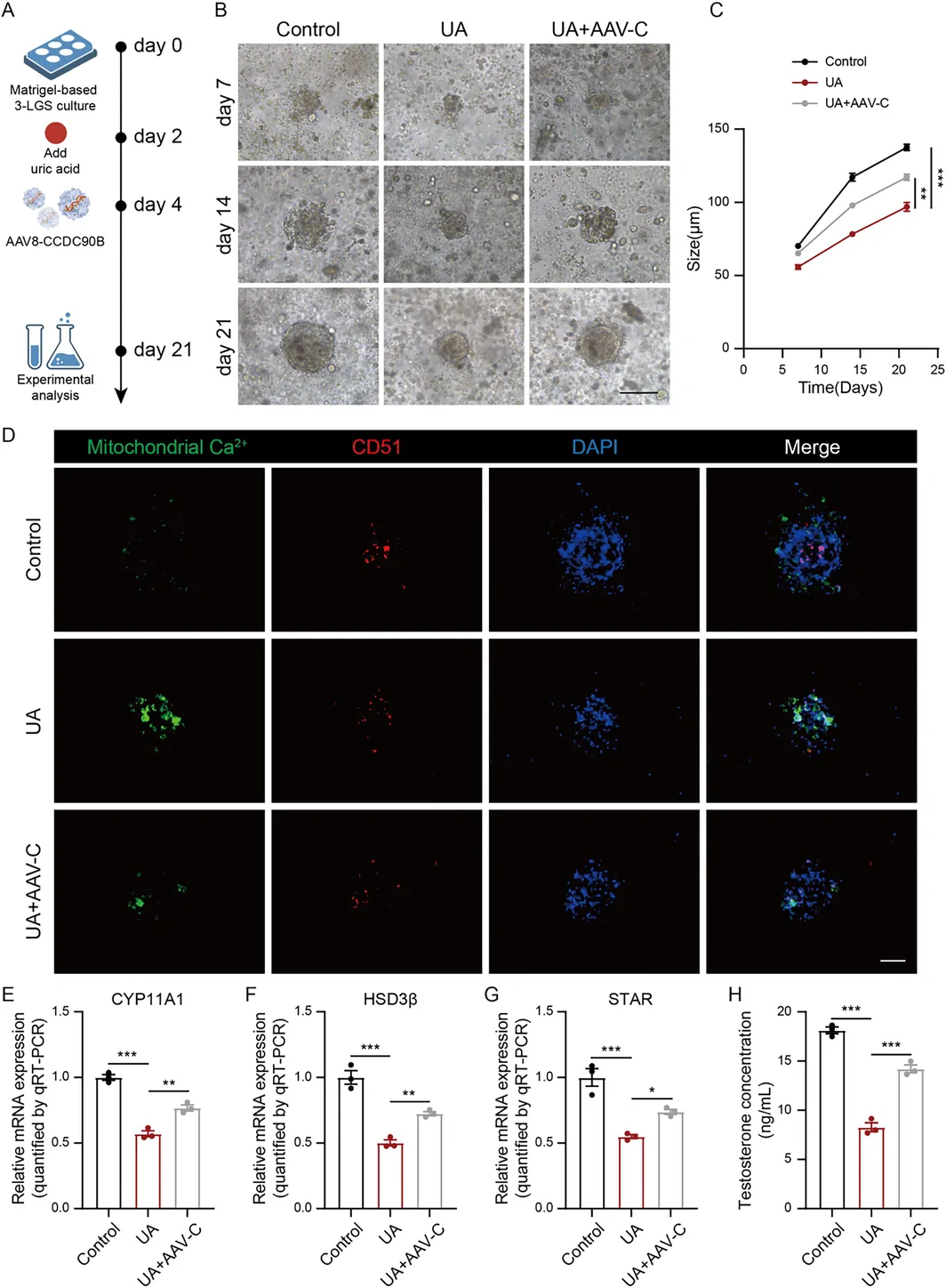
Targeting CCDC90B rescues testicular organoids from high uric acid‐induced inhibition via mitochondrial quality control. (A) Schematic diagram of the experimental process related to testicular organoids. (B) Representative images of testicular organoids cultured in matrigel with uric acid and uric acid + AAV‐CCDC90B (C) for 21 days (bar = 100 μm). (C) Quantitative analysis of average value of testicular organoids diameter (μm) in (B). (*n* = 3 biological repeats for each group). (D) Representative immunofluorescence microscopy images of testicular organoids mitochondrial calcium ion and CD51, cultured in matrigel with uric acid and uric acid + AAV‐C (bar = 100 μm). (E–G) qPCR analysis of relative mRNA expression of LC markers in testicular organoids of Control, UA and UA + AAV‐C groups. (H) Measurement of testosterone concentration in the medium of testicular organoids. Data are represented as mean ± SEM. In all bar graphs, each dot represents one biological replicate. *p < 0.05, **p < 0.01, ***p < 0.001; ns, no significance by one‐way ANOVA (C, and E–H).

### 2.7 AAV8‐Mediated Downregulation of CCDC90B Increases Testosterone Levels and Diminishes the Effect of High UA in Promoting SLC Ageing

To further validate our in vitro findings, we knocked down CCDC90B expression in SLCs via interstitial injection of AAV8 and verified key experimental conclusions. Fluorescence imaging shows that AAV8 can specifically target the testicular interstitium (Figure S7A). H&E staining revealed that, compared to the Uox^−/−^ group, the testicular interstitial area was increased in the Uox^−/−^ + AAV‐C group (Figure 7A,B). Quantitative analysis showed a significant increase in the number of LCs and the expression of LC‐related markers in the Uox^−/−^ + AAV‐C group (Figure 7C–F). Concurrently, serum testosterone and testicular testosterone levels in the Uox^−/−^ + AAV‐C group were markedly higher than those in the Uox^−/−^ group (Figures 7G and S7B). Regarding MQC, relevant assays indicated that AAV8‐targeted CCDC90B interference reduced mitochondrial calcium content and ROS levels in the SLCs of Uox^−/−^ mice (Figure S7C–F). Additionally, mitochondrial membrane potential and ATP levels were significantly elevated in the SLCs of Uox^−/−^ mice following treatment (Figure S7G–I). In terms of SLC senescence, relevant findings showed that compared to the Uox^−/−^ group, the Uox^−/−^ + AAV‐C group exhibited fewer SA‐β‐gal‐positive SLCs, increased p‐RB levels and downregulated expression of p16, p21, p53 and SASP factors (Figure 7H–L). Collectively, these results indicate that targeting CCDC90B expression via AAV8 alleviates SLC senescence and elevates testosterone levels in Uox^−/−^ mice.

**FIGURE 7:**
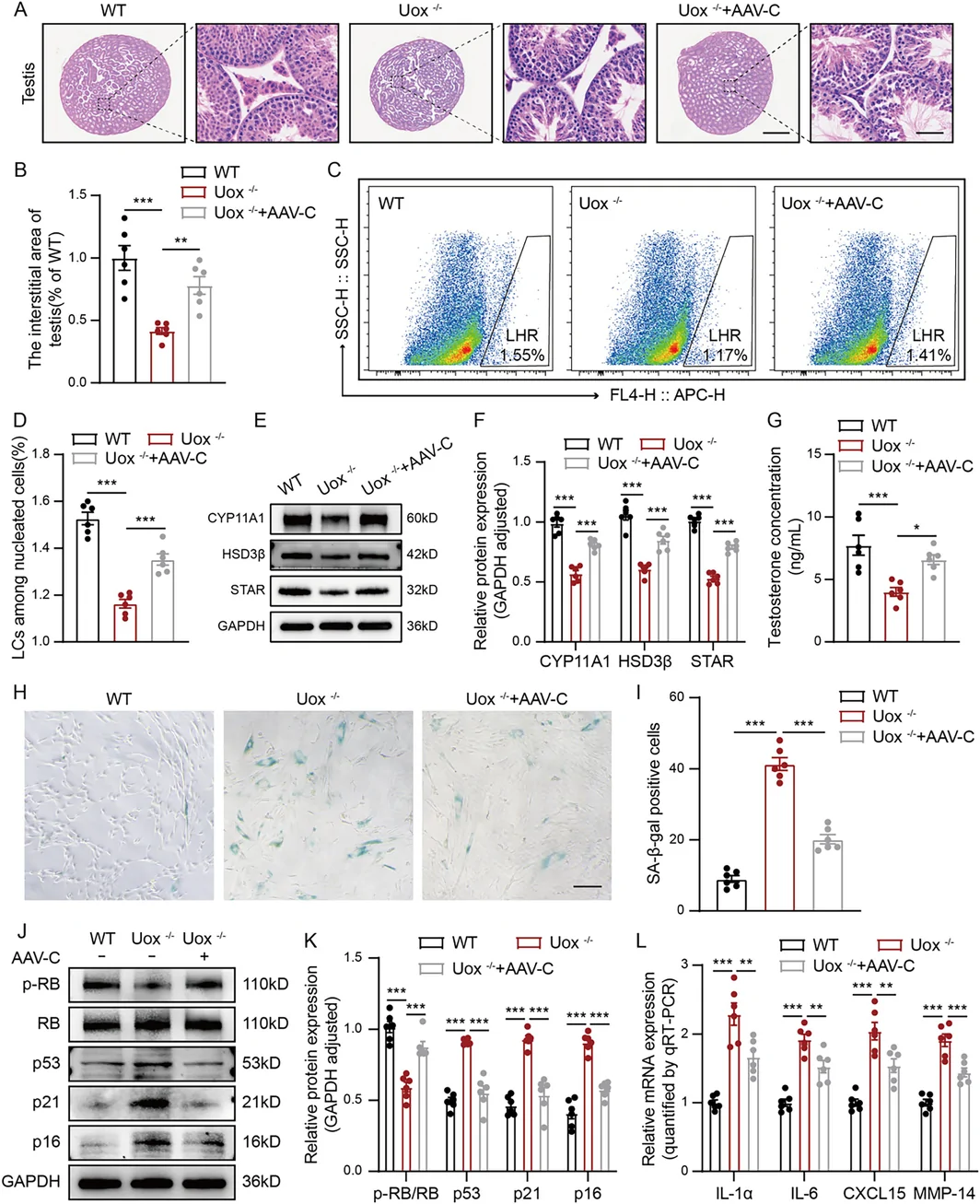
AAV‐mediated downregulation of CCDC90B increases testosterone levels and diminishes the effect of high uric acid in promoting SLC ageing. (A) Representative H&E staining pictures of testicular mesenchyme from WT, Uox^−/−^, Uox^−/−^ + AAV‐CCDC90B (C) groups (*n* = 6 biological repeats for each group; low power lens: Bar = 500 μm, high power lens: bar = 50 μm). (B) Quantification of mean area of the testicular interstitium in (A). (C) Flow cytometry for detecting the percentage of LHR^+^cells in the testis of WT, Uox^−/−^, Uox^−/−^ + AAV‐C groups. (D) Quantification of percentage of LHR^+^ cells in the testis of WT, Uox^−/−^, Uox^−/−^ + AAV‐C groups in (C). (E, F) Western Blot analysis and quantification of LC markers' expression in SLCs of WT, Uox^−/−^, Uox^−/−^ + AAV‐C groups. (G) Measurement of testosterone concentration in the serum. (H, I) SA‐β‐Gal images and quantification analysis showed the number of SA‐β‐Gal‐positive cells in SLCs. (J, K) Western Blot analysis and quantification of senescence markers' expression in SLCs of WT, Uox^−/−^, Uox^−/−^ + AAV‐C groups. (L) qPCR analysis of relative mRNA expression of the SASP genes in SLCs of WT, Uox^−/−^, Uox^−/−^ + AAV‐C groups. Data are represented as mean ± SEM. In all bar graphs, each dot represents one biological replicate. *p < 0.05, **p < 0.01, ***p < 0.001; ns, no significance by one‐way ANOVA (B, D, F, G, I, K and L).

Based on the aforementioned data, high UA induces SLC senescence. Importantly, CCDC90B plays an indispensable role in this process, likely by inducing MQC imbalance via mitochondrial calcium overload (Graphical Abstract).

## 3 Materials and Methods

### 3.1 Ethics Statement

The mouse experiments were carried out according to protocols approved by the Institutional Animal Care and Use Committee (ACUC) of the Shenzhen Top Biotech. Co. Ltd. (Approval number: TOPGM‐IACUC‐2024‐0179). The male wildtype (WT) and Uox^−/−^ mice used in this study were derived from a breeding colony of Uox heterozygous (Uox^+/−^) C57BL/6J mice, was generated using CRISPR/Cas9 technology by GemPharmatech Co. Ltd. (Nanjing, China). Genotyping of the mice was performed using PCR on DNA extracted from tail samples. Uox knockout primer sequences are as follows: forward primer, 5′‐ATCTCCTCCCTGGCAGCAAACT‐3′ and reverse primer, 5′‐GAGTCTCTTATGCAGATAGAAGTGTTGC‐3′. 12‐week‐old WT and Uox^−/−^ mice were randomly assigned to experimental groups. Mice were housed in the Top Biotech. Co. Ltd. (Shenzhen, China) under specific pathogen‐free (SPF) conditions at 23°C ± 2°C ambient temperature with 40% humidity and a 12 h light/dark cycle.

### 3.2 Cell Lines and Primary Cell Culture

Isolation and culture of primary mouse SLCs were performed as follows: the testes were dissected from WT and Uox^−/−^ mice and the tunica albuginea was carefully removed, after which the testes were minced into small pieces. Interstitial cells were dissociated from seminiferous tubules using 1 mg/mL collagenase type IV (Gibco) in Dulbecco's modified Eagle's medium (DMEM)/F12 (Gibco) at 37°C for 15 min. The supernatant was filtered through a 45 μm strainer and collected and the cells in the supernatant were subsequently acquired by centrifuging at 500*g* for 5 min. For in vivo experiments, the mixed cells were re‐suspended in PBS. The CD51^+^cells were enriched by FACS using an Influx Cell Sorter (Becton Dickinson), after which they were seeded in SLCs culture medium. For in vitro experiments, the mixed cells were washed twice with PBS, re‐suspended in PBS and seeded in SLCs culture medium. The medium consisted of DMEM/F12 supplemented with 1 nM dexamethasone (Sigma‐Aldrich), 1 ng/mL LIF (Millipore), 5 μg/L insulin‐transferrin‐sodium selenite (Sigma‐Aldrich), 5% chicken embryo extract (US Biologicals), 0.1 mM β‐mercaptoethanol (Invitrogen), 1% nonessential amino acids (HyClone), 1% N2 (Invitrogen) and 2% B27 (Invitrogen) supplements, 20 ng/mL basic fibroblast growth factor (Invitrogen), epidermal growth factor (PeproTech), platelet‐derived growth factor (PeproTech) and oncostatin M (PeproTech). SLCs were maintained at 37°C and 5% CO_2_ in a humidified atmosphere and confirmed to be mycoplasma‐free. The medium is changed every 3 days.

### 3.3 Construction of Testicular Organoids

The testicular tissue of 8‐week‐old mouse was minced (1 mm^3^) and the primary testicular cells (including testicular somatic cells and germ cells) were collected by enzymatic digestion. The primary testicular cells were resuspended in matrigel and grown in the three‐layer gradient system (3‐LGS), as described in the reference [45]. In brief, drop 10 μL matrigel onto a petri dish and place it upside down in the incubator to allow it to solidify. Then, drop 6 μL matrigel containing primary cells onto the solidified matrigel and place it upside down to allow it to solidify. Finally, drop 16 μL matrigel onto the matrigel containing primary cells, let it solidify and then add the culture medium. Testicular organoids were cultured in MEM‐α (Gibco) with 10% KSR (Gibco), 1% penicillin and streptomycin (Gibco), for 21 days at 37°C, with medium changes every 48 h.

### 3.4 Measuring Organoid Formation

Representative bright‐field images (×20 magnification) were acquired every seven days. Fiji software was used to measure the diameter of the generated organoids and to plot growth curves.

### 3.5 Single‐Cell RNA Sequencing Library Preparation and Sequencing

Single‐cell RNA‐seq libraries were constructed using the SeekOne Digital Droplet Single Cell 3′ Library Preparation Kit (SeekGene), following the manufacturer's instructions. In brief, a defined number of single cells were mixed with reverse transcription reagents and loaded into the sample wells of the SeekOne microfluidic chip. Barcoded Hydrogel Beads (BHBs) and partitioning oil were then added into designated wells to generate emulsified droplets. Reverse transcription was carried out within the droplets at 42°C for 90 min, followed by enzyme inactivation at 80°C for 15 min. Subsequently, cDNA was extracted from the broken emulsions, purified and subjected to PCR amplification. The amplified cDNA was further processed by fragmentation, end repair, A‐tailing and ligation with sequencing adapters. Indexed PCR amplification was then performed to enrich the 3′ end of transcripts, incorporating both cell barcodes and unique molecular identifiers (UMIs). The final libraries were purified using SPRI beads, quantified using qPCR (KAPA Biosystems) and sequenced on an Illumina NovaSeq 6000 platform with paired‐end 150 bp reads. Seurat (version 4.1.3) was used to perform the data processing. After quality control, normalisation, cell clustering and cell type annotation, we performed the following analyses: compositional analysis, differential expression testing, gene set variation and gene set enrichment.

### 3.6 AAV8 for SLCs

AAV8 vectors, encompassing the shRNA of mouse CCDC90B, were procured from Vigene Bioscience, along with vectors encoding mCherry. For in vivo administration, either phosphate‐buffered saline (PBS) or AAV particles were injected into the interstitial space of individual testis of 8‐week‐old male mice. Mice were anaesthetised and intra‐testis administered with PBS or AAV8 at doses of 8 × 10^10^ genome copies (gc)/testis. Conduct the corresponding experimental tests 4 weeks after the injection. For in vitro administration, Dulbecco's Modified Eagle Medium/Nutrient Mixture F‐12 (DMEM/F12) or AAV particles were added to culture medium at doses of 8 × 10^11^ gc/organoid. The corresponding experiments in organoid were tested 3 weeks post‐intervention.

### 3.7 Leydig Cell Differentiation and LC Markers Fluorescence Detection

When SLC proliferation reached about 80%, fresh differentiation‐inducing medium was replaced and they were incubated for 7 days, as previously described [47]. The medium consisted of phenol red‐free DMEM/F12 and M199 medium (1:1; Gibco) supplemented with 2% FBS, 10 ng/mL PDGFAA (PeproTech), 1 ng/mL LH (R&D Systems), 250 nM Smoothened Agonist HCl (SAG; Millipore), 1 μM Forskolin (Fsk; Sigma‐Aldrich), 50 ng/mL insulin‐like growth factor 1 (IGF1, PeproTech) and 5 μg/L insulin‐transferrin‐sodium selenite (Sigma‐Aldrich) and they were incubated for 14 days. For the fluorescence detection of LC markers. Cells were fixed, penetrated and blocked, then incubated with the primary antibodies and secondary antibodies. Randomly sample the field of view and uniformly determine the cell density. Data are presented as mean fluorescence intensity (MFI) and analysed using Fiji software.

### 3.8 Drug Affinity Responsive Target Stability

The DARTS assay was conducted following the method described by Lomenick et al. [48], with slight modifications. In brief, SLCs were seeded in 10 cm^2^ culture dishes and cultured until reaching approximately 80% confluence. Total proteins were extracted using the Mammalian Protein Extraction Reagent (M‐PER) supplemented with protease and phosphatase inhibitors, according to the manufacturer's instructions. After centrifugation at 13,000 rpm for 15 min, the supernatants were collected. UA (100 μM) or an equivalent volume of DMSO was added to the protein extracts and incubated for 30 min. Proteolysis was then carried out, during which drug‐bound proteins exhibited increased resistance to protease digestion. All procedures were performed on ice to minimise protein degradation. Protein bands were excised from the gels and subjected to in‐gel trypsin digestion for subsequent mass spectrometry analysis. Peptide samples were analysed by LC–MS/MS using a Thermo LTQ‐Orbitrap mass spectrometer. For quantitative analysis of protein and peptide levels, MS data were processed using the differential analysis workflow of Rosetta Elucidator (Rosetta Inpharmatics) [49].

### 3.9 H&E Staining

The testes of mice were obtained and fixed in 4% paraformaldehyde (PFA), then embedded in paraffin. The embedded tissues were sectioned, dewaxed to water and stained with haematoxylin and eosin (H&E).

### 3.10 Testosterone Concentration Assay

Collect mouse serum and cell culture medium for testosterone quantitative detection. Testosterone levels were measured using a commercially available ELISA kit (Fine Biotech) according to the manufacturer's instructions.

### 3.11 Draw the Fitting Curve of IC50

Set up a culture environment with 1, 2, 3, 4 and 5 mM UA. Cell viability was measured via a CCK8 kit (Yeasen) according to the manufacturer's instructions. A microplate reader was used to detect the absorbance at 450 nm and calculated the survival rate of cells.

### 3.12 Flow Cytometric Assay

Cut the tissue into small pieces to obtain the testicular cells and then wash them three times with FACS (1% bovine serum albumin in PBS containing 0.5 mM EDTA) buffer solution. The cells were incubated with primary and secondary antibodies under a dark environment at 4°C. After the incubation, the cells were washed twice with FACS buffer and run on CytoFLEX (Beckman Coulter, USA).

### 3.13 Immunofluorescence Staining

Cells, testicular tissue sections and testicular organoids were fixed, penetrated and blocked, then incubated with the primary antibodies overnight at 4°C, followed by incubation with secondary antibodies away from light for 1 h at room temperature. The primary and secondary antibodies used were described in Table S1. Images were acquired using LSM800 confocal microscope (Zeiss) or DMI8 (Leica).

### 3.14 SA‐β‐Gal Staining

According to the manufacturer's protocol of senescence‐associated β‐galactosidase kit (Beyotime, C0602), remove the cell culture medium from the 24‐well plate, wash twice with PBS and then add 4% PFA for fixation for 20 min. After fixation, the sample was washed twice with PBS and then the working solution of β‐galactosidase plus X‐Gal was added and incubated at 37°C overnight. The next day, observations were made under an optical microscope (Leika, DMi8) and counts were taken from three randomly selected fields.

### 3.15 Clonal Sphere Formation Assay

SLCs resuspended into 12‐well plates at a density of 10,000 cells/mL for daily observation. Spheres were defined as free‐floating spherical structures with a diameter > 50 μm. Subsequently, primary spheres (Day 10) were dissociated into single cells and replated under the same culture conditions as those used for the growth of primary spheres to generate secondary spheres. All spheres in the Wells were counted and diameters measured.

### 3.16 Measurements of Cell ROS and Mitochondrial ROS

Cell ROS and mitochondrial ROS levels are detected using the fluorescent dye CellROX Green and MitoSOX Red (Invitrogen). Following the manufacturer's protocol, cells are incubated in medium containing fluorescent dye for 30 min. After washing, labelled cells are evaluated using flow cytometry. Data are analysed using FlowJo v10.6.2 software.

### 3.17 Measurements of Mitochondrial Calcium Ion

Mitochondrial calcium ion was detected by adding 1X Rhod‐2 AM (Beyotime) to the medium and incubated at 37°C for 20 min. After incubation, the supernatant was removed and the sample was washed twice with PBS. Then, an appropriate amount of PBS was added and the sample was observed under a fluorescence microscope. Calcium images were obtained using the fluorescence microscope DMI8 (Leica); the average fluorescence intensity was assessed with Fiji software.

### 3.18 Transmission Electron Microscopy

Cell samples were fixed in 2.5% glutaraldehyde for 2 h at room temperature and then stored at 4°C overnight. Subsequently, samples were post‐fixed with 1% osmium tetroxide for 1 h, washed, dehydrated through an ethanol gradient (30%, 50%, 70% and 95%, 5 min per step), embedded and polymerised at 60°C for 48 h. Ultrathin sections of 80 nm were cut and observed in a Tecnai 12 BioTwin transmission electron microscope (FEI Company, Eindhoven, The Netherlands) at 120 keV.

### 3.19 Measurement of Mitochondrial Membrane Potential (MMP)

MMP was measured using an enhanced mitochondrial membrane potential assay kit with JC‐1 (Beyotime). According to the manufacturer's instructions, SLCs were incubated in the JC‐1 staining solution at 37°C for 20 min, then washed with the JC‐1 staining buffer and immersed in the culture medium. The normal mitochondria react with JC‐1 aggregates to produce red fluorescence, while the depolarised mitochondria react with JC‐1 monomers to produce green fluorescence. The fluorescence intensity is quantified using a flow cytometer. The data are analysed using FlowJo v10.6.2 software and the ratio of red to green fluorescence intensities is calculated.

### 3.20 Measurement of Mitochondrial ATP

Following the manufacturer's instructions for the ATP Assay Kit (Beyotime), after centrifugation, remove the cell debris and add the supernatant to the substrate solution. Record the luminescence of the solution in an illuminometer, with each well's luminescence measured for 10 s. Use the BCA Protein Assay Kit (Beyotime) to determine the protein content and then convert the ATP concentration to micromole per milligramme of protein.

### 3.21 RNA Isolation and Real‐Time Quantitative PCR (qPCR)

Total RNA was extracted from testis tissue or cells using the TRIzol reagent (Thermofisher Scientific) according to the manufacturer's protocol. Quantification was performed with a NanoDrop 8000 spectrophotometer and 1 μg of total RNA was used for reverse transcription with the HiFiScript All‐in‐one RT Master Mix Kit (cwbiotech). cDNAs were used as the template for qPCR reactions with the SuperStar Universal SYBR Master Mix (cwbiotech). All samples were run in triplicate and the results were normalised to 18 S rRNA as relative mRNA levels. The primers designed and used for qPCR are described in Table S2.

### 3.22 Western Blot

Cell suspensions were collected and washed three times with PBS. Then, the cells were lysed in 1X RIPA buffer with 1X protein inhibitor solution for at least 30 min and centrifuged at 12,000*g* for 10 min at 4°C to remove cell debris. The protein supernatant was aspirated and mixed with 4X loading buffer (Bio‐RED). After total protein concentration was measured using BCA Protein Assay Kit (Beyotime), equal amounts of total proteins were resolved by SDS‐PAGE (Epizyme) and then electrotransferred to a 0.45 μm pore‐sized polyvinylidene difluoride membrane (Millipore). After blocked with 5% BSA, the membrane was incubated with the primary antibodies at 4°C overnight, followed by incubation with HRP‐conjugated secondary antibodies for 1 h at room temperature. The primary and secondary antibodies used can be found in Table S1. The chemiluminescent substrate (Beyotime) was used to detect the signal intensity. Bands from at least three independent blots were quantified using the Fiji software.

### 3.23 Co‐Immunoprecipitation (Co‐IP)

SLCs were incubated on ice for 5 min with 300 μL lysis buffer (Beyotime) (1 mM PMSF, protease inhibitor and phosphatase inhibitor). The cells were scrape‐harvested, cellular debris was removed by centrifugation for 10 min at 12,000 rpm at 4°C and the concentration of protein was determined. Cell supernatants were incubated with primary antibody overnight at 4°C, followed by addition of 50 μL protein G agarose beads (Santa Cruz Biotechnology) for 2 h at 4°C. Immunoprecipitates were washed two times with lysis buffer, separated by centrifugation for 2 min at 5000 rpm and then heated with 5× sample buffer for electrophoresis and western blot analysis.

### 3.24 Statistical Analyses

All experiments were carried out with at least three biological replicates and successful reproducibility was shown. All data are reported as the mean ± standard error of mean (SEM) of at least three independent experiments. Sample sizes are all presented in the figure legends. Statistical analysis between two groups was performed using an unpaired *t*‐test. Statistical analysis between multiple groups was performed by one‐way ANOVA. All data were analysed using GraphPad Software. A two‐sided *p*‐value < 0.05 was considered to be statistically significant. The level of significance defined as *p < 0.05, **p < 0.01, ***p < 0.001.

## 4 Discussion

Currently, extensive research has demonstrated a close association between metabolic diseases and impaired male reproductive function [50, 51, 52]. Studies have reported the effects of hyperlipidemia and diabetes on male reproductive functions [13, 14]. However, the role of hyperuricemia in male reproductive dysfunction remains unclear. In this study, we identified a hyperuricemic microenvironment as a key driver of SLC senescence. Mechanistically, excessive UA binds to the mitochondrial inner membrane protein CCDC90B in SLCs and inhibits its degradation, leading to its abnormal accumulation, mitochondrial calcium overload and subsequent MQC imbalance, thereby inducing SLC senescence. Importantly, targeted knockdown of CCDC90B significantly alleviates SLC senescence. These findings identify CCDC90B as a key therapeutic target for treating low testosterone levels induced by hyperuricemia.

Cellular senescence is characterised by progressive functional decline and is closely linked to disrupted stem cell homeostasis [53, 54]. Stem cells drive tissue regeneration through proliferation and differentiation, but these capacities decline with ageing [55, 56]. For example, haematopoietic stem cells exhibit reduced proliferation and regenerative capacity [56, 57, 58], mesenchymal stem cells shift towards adipogenic differentiation contributing to osteoporosis [59] and hair follicle stem cell senescence leads to follicle miniaturisation and alopecia [60]. Our previous studies have shown that SLCs maintain the regenerative capacity of the testicular interstitium and support testosterone synthesis and secretion through their population size and differentiation potential [61]. However, we observed SLC senescence in hyperuricemic mice, resulting in insufficient LC replenishment and reduced testosterone levels. Distinct from natural ageing, hyperuricemia‐induced premature senescence suggests that metabolic stress directly drives stem cell dysfunction. Therefore, elucidating how a hyperuricemic microenvironment promotes SLC senescence is critical for addressing UA‐induced testosterone deficiency.

Mitochondrial homeostasis is essential for stem cell function, whereas its disruption leads to excessive ROS accumulation and cellular damage [62, 63]. MQC maintains this balance, and its dysfunction results in mitochondrial abnormalities, ROS overproduction and impaired stem cell function [64, 65]. In this context, ROS‐mediated signalling, including Wnt and Hedgehog pathways, may further contribute to altered stem cell fate [66, 67, 68]. For instance, BNIP3 depletion in embryonic stem cells results in mitochondrial dysfunction and impaired differentiation [69], while disruption of Mfn1/2 or OPA1 affects neural stem cell self‐renewal via ROS‐mediated signalling [70]. In addition, MQC imbalance contributes to neuronal ferroptosis, whereas MSC‐mediated mitochondrial transfer can restore mitochondrial function [64]. Our data show that high UA disrupts MQC in SLCs, as evidenced by increased mitochondrial ROS, reduced membrane potential, altered morphology and defective mitophagy. These changes are accompanied by downregulation of MQC regulators and antioxidant enzymes, exacerbating oxidative stress and promoting SLC senescence. Together, these findings support an MQC‐dependent mechanism of SLC senescence and reveal a novel organelle‐level mechanism of mitochondria.

CCDC90B is a coiled‐coil domain protein with a conserved α‐helical structure that mediates protein–protein interactions and mitochondrial localisation. It associates with ubiquitinated proteins and mitochondrial functional components, contributing to mitochondrial structural organisation and quality control [36]. As a key mitochondrial structural protein, CCDC90B is essential for maintaining mitochondrial integrity and homeostasis [71]. Although previous studies have suggested that CCDC90B is involved in mitochondrial dynamics and energy metabolism, its role in MQC and reproductive biology remains unclear. Using DARTS, we identified CCDC90B as a UA‐binding target in SLCs. Its abnormal accumulation induces mitochondrial calcium overload and disrupts MQC, revealing a previously unrecognised role in mitochondrial homeostasis. Importantly, AAV‐mediated targeting of CCDC90B effectively alleviates SLC senescence under hyperuricemic conditions, highlighting its potential as a therapeutic strategy.

Organoids are three‐dimensional culture systems that recapitulate key architectural organisation, multicellular composition and niche signalling of native tissues, thereby preserving cell–cell interactions and local microenvironmental cues more effectively than conventional 2D cultures [44]. In the testicular field, their utility has been demonstrated in reconstructing seminiferous tubule–like structures and spermatogenic niches [45] and enabling functional evaluation of gene therapy strategies such as AAV‐mediated rescue of steroidogenic defects [46]. Despite limitations, including incomplete vascularisation and lack of systemic endocrine regulation, testicular organoids represent a highly valuable experimental model.

However, certain limitations persist in our current exploration of the hyperuricemic microenvironment and research into SLC‐based therapies. First, the molecular mechanism by which UA interferes with CCDC90B ubiquitination requires further clarification. Second, it is necessary to further validate the mechanistic role of CCDC90B in other metabolic disease microenvironment models, such as high‐fat and diabetic models. Third, LCs are the main cells involved in testosterone synthesis. The impact of high UA on the LCs' function remains to be clarified and this is the work that we urgently need to do next. Finally, its therapeutic efficacy needs confirmation in clinical settings to support translational applications.

This study represents a significant advance, further establishing that SLC senescence is a major driver of hyperuricemia‐induced testosterone deficiency. Among the key regulators of SLC senescence, we demonstrate that UA‐induced accumulation of CCDC90B disrupts MQC, thereby promoting SLC senescence. Notably, these findings position CCDC90B and mitochondrial calcium homeostasis as critical nodes linking systemic metabolic disturbances to testicular ageing. By connecting SLC senescence with mitochondrial homeostasis and proposing CCDC90B inhibition as a strategy to restore functional SLCs and testosterone production, this study provides new insights into testicular endocrine regulation. Moreover, targeting the UA–CCDC90B axis may represent a promising therapeutic approach not only for hyperuricemia‐associated hypogonadism but also for broader age‐related declines in steroidogenic function. In summary, our findings uncover a novel MQC‐dependent mechanism of SLC senescence and support the clinical translation of precision AAV‐based SLC‐targeted therapies for mitochondrial‐related testicular endocrine disorders.

## Author Contributions

**Jiayu Huang:** conceptualisation, methodology, data curation, formal analysis, investigation, writing – original draft, funding acquisition. **Ao Wang:** data curation, formal analysis, investigation. **Xiangyu Li:** data curation, formal analysis, investigation. **Peng Huang:** methodology. **Kaixuan Zeng:** investigation, writing – original draft. **Lu Sun:** investigation. **Moxuan Li:** investigation. **Yixiang Chen:** investigation. **Jiancheng Wang:** conceptualisation, writing – review and editing, funding acquisition, resources, supervision.

## Funding

The funding for this project was provided by the National Natural Science Foundation of China (82371608 and 32570940); Guangdong Basic and Applied Basic Research Foundation (2023B1515020016); Shenzhen Science and Technology Program (Grants JCYJ20240813150422030 and JCYJ20250604143318024); Research Start‐up Fund of the Seventh Affiliated Hospital, Sun Yat‐sen University (ZSQYRSSFAR0003).

## Conflicts of Interest

The authors declare no conflicts of interest.

## Supporting information


**Figure S1:** Testicular weight and LC count decrease in the mice with hyperuricemia.
**Figure S2:** SLCs show upregulation of ageing‐related genes in the hyperuricemia mice.
**Figure S3:** High uric acid environment drives SLCs to express SASP factor and reduces steroid synthesis.
**Figure S4:** SLCs exhibit mitochondrial quality control imbalance under high uric acid conditions.
**Figure S5:** Interference with CCDC90B maintains mitochondrial quality control of SLCs under high uric acid conditions.
**Figure S6:** AAV‐mediated downregulation of CCDC90B reduces the expression of ageing‐related genes in testicular organoids under high uric acid environment.
**Figure S7:** Interference with CCDC90B maintains mitochondrial quality control of SLCs in the hyperuricemia mice.
**Table S1:** Antibodies, chemical reagents and kits.
**Table S2:** Primers used for real‐time PCR.

## Data Availability

The data that support the findings of this study are available from the corresponding author upon reasonable request.
