# Supplementary material for: High Uric Acid Promotes Stem Leydig Cell Senescence by CCDC90B Mediates Mitochondrial Quality Control Imbalance
Source: Cell Prolif. 2026 Jun 4:e70237. Online ahead of print. doi: 10.1111/cpr.70237 (PMC13325638; doi:10.1111/cpr.70237)
Supplement: Supplementary file 1 — Figure S1: Testicular weight and LC count decrease in the mice with hyperuricemia. Figure S2: SLCs show upregulation of ageing‐related genes in the hyperuricemia mice. Figure S3: High uric acid environment drives SLCs to express SASP factor and reduces steroid synthesis. Figure S4: SLCs exhibit mitochondrial quality control imbalance under high uric acid conditions. Figure S5: Interference with CCDC90B maintains mitochondrial quality control of SLCs under high uric acid conditions. Figure S6: AAV‐mediated downregulation of CCDC90B reduces the expression of ageing‐related genes in testicular organoids under high uric acid environment. Figure S7: Interference with CCDC90B maintains mitochondrial quality control of SLCs in the hyperuricemia mice. Table S1: Antibodies, chemical reagents and kits. Table S2: Primers used for real‐time PCR. [file CPR-9999-e70237-s001.docx]

**Supplemental Information**

**High uric acid promotes stem Leydig cell senescence by CCDC90B mediates mitochondrial quality control imbalance**

**Huang et al.**


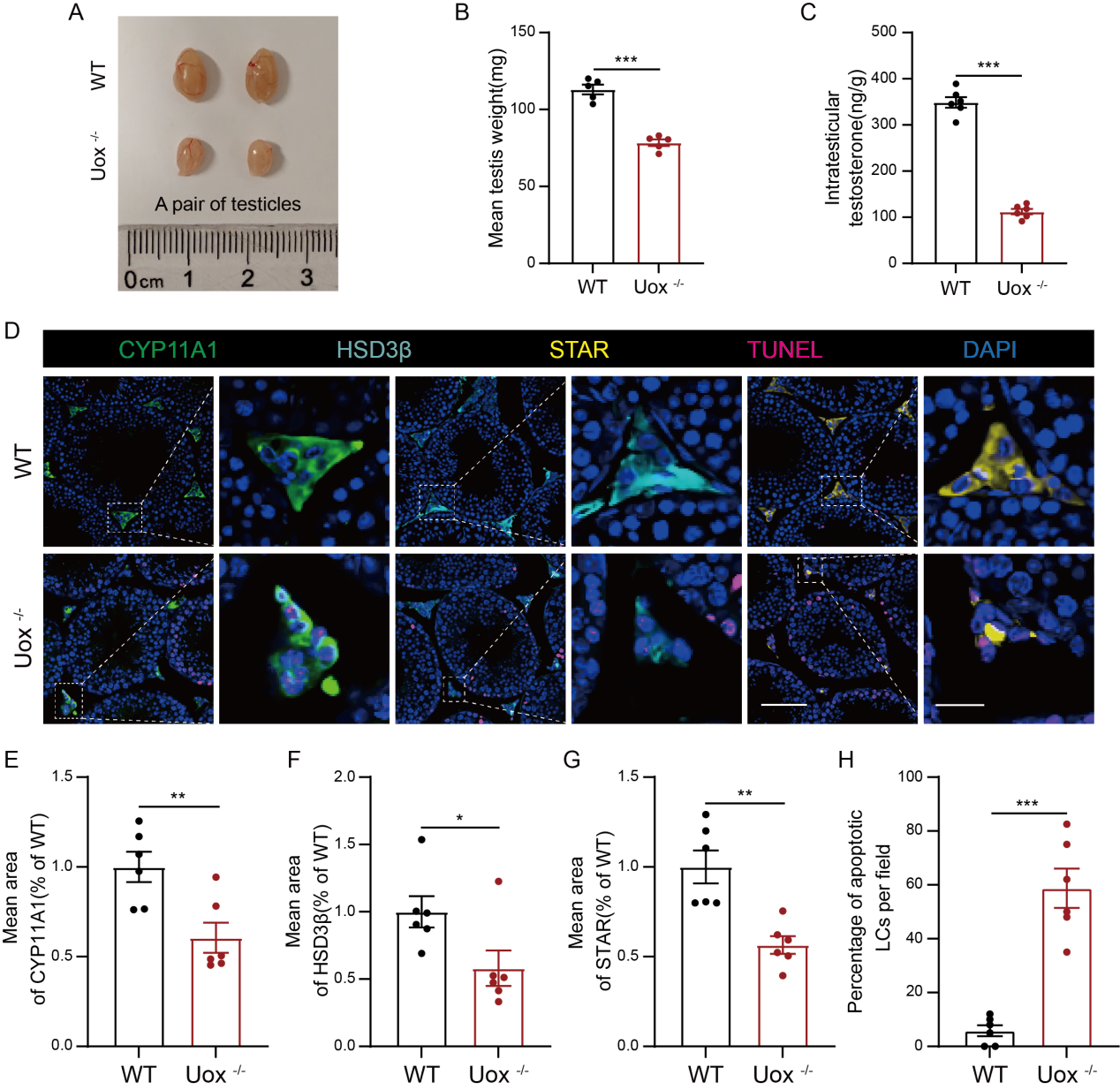


**Figure S1 | Testicular weight and LC count decrease in the mice with hyperuricemia.**

(A) Representative images of testes of WT and Uox^⁻/⁻^ mice.

(B) Quantification of mean weight of the testis in (A).

(C) Measurement of testosterone concentration in the testis.

(D) Representative immunofluorescence images of LC markers and TUNEL in testis frozen sections of WT, Uox^⁻/⁻^, Uox^⁻/⁻^ + AAV-C groups. (n = 6 biological repeats for each group; low power lens: bar = 100 μm, high power lens: bar = 50 μm).

(E-G) Quantification of mean fluorescence area of LC markers in (D).

(H) Quantification of percentage of apoptotic LCs in (D).

Data are represented as mean ± SEM. In all bar graphs, each dot represents one biological replicate. *p < 0.05, **p < 0.01, ***p < 0.001; ns, no significance by unpaired Student’s t test (B, C and E-H).


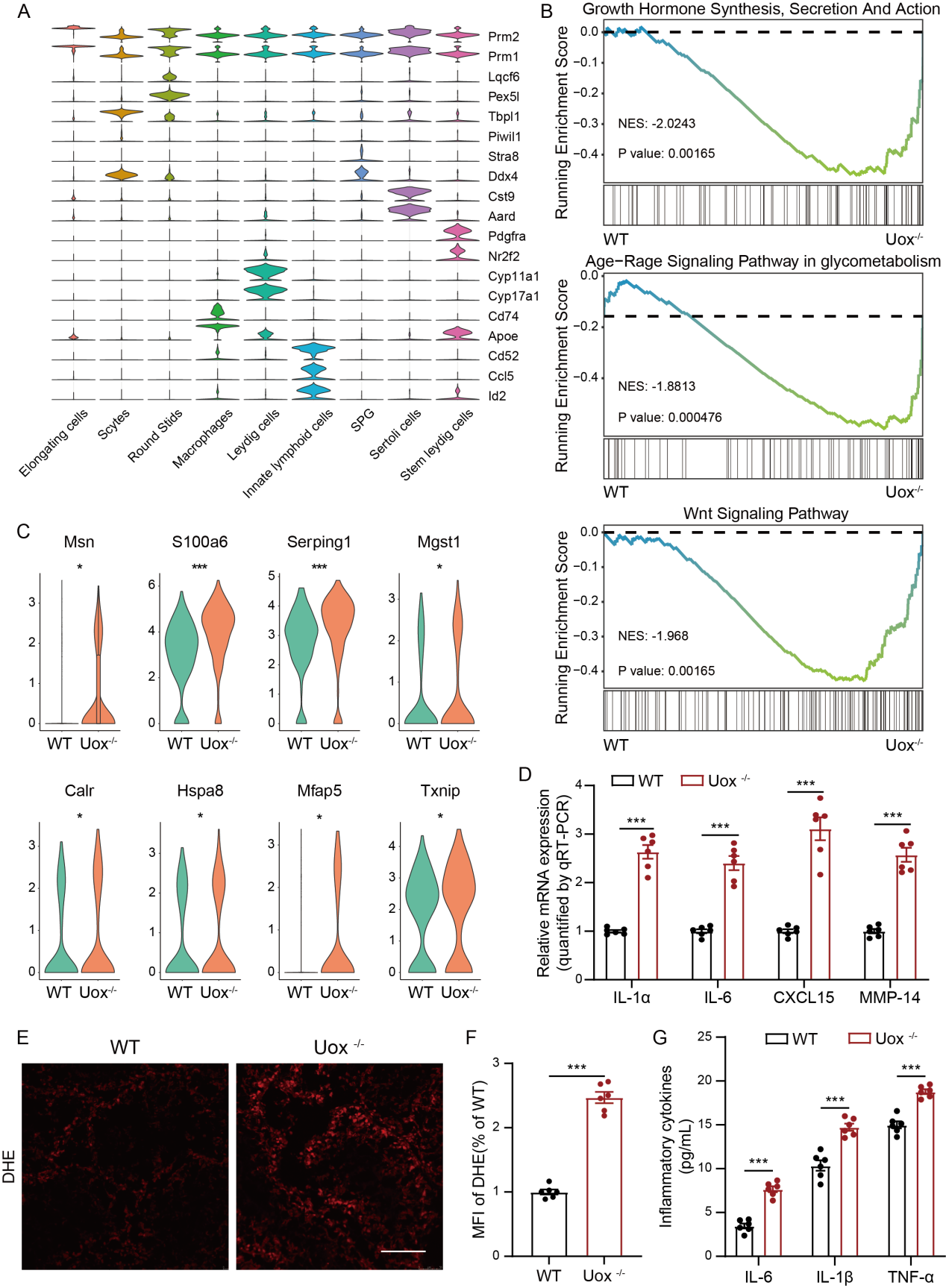


**Figure S2 | SLCs show upregulation of aging-related genes in the hyperuricemia mice.**

(A) The bubble plot of normalized mean expression for key signature genes for each

major cell type in **Fig. 2A**.

(B) GSEA of the hallmark gene sets in the MSigDB database revealing the enrichment of Aging-related pathways GO terms in SLCs. NES, normalized enrichment score.

(C) Expression levels of Aging-related genes in SLCs from normal and hyperuricemia testis.

(D) qPCR analysis of relative mRNA expression of the SASP genes in SLCs of WT and Uox^⁻/⁻^ groups.

(E) Fluorescent staining of testis slices with Dihydroethidium (DHE) to detect ROS. (bar = 100 μm).

(F) Quantification of mean fluorescence intensity of DHE in (E).

(G) ELISA experiment for detecting the content of IL-6, IL-1β and TNF-α in testes.

Data are represented as mean ± SEM. In all bar graphs, each dot represents one biological replicate. *p < 0.05, **p < 0.01, ***p < 0.001; ns, no significance by unpaired Student’s t test (B, D, F and G).


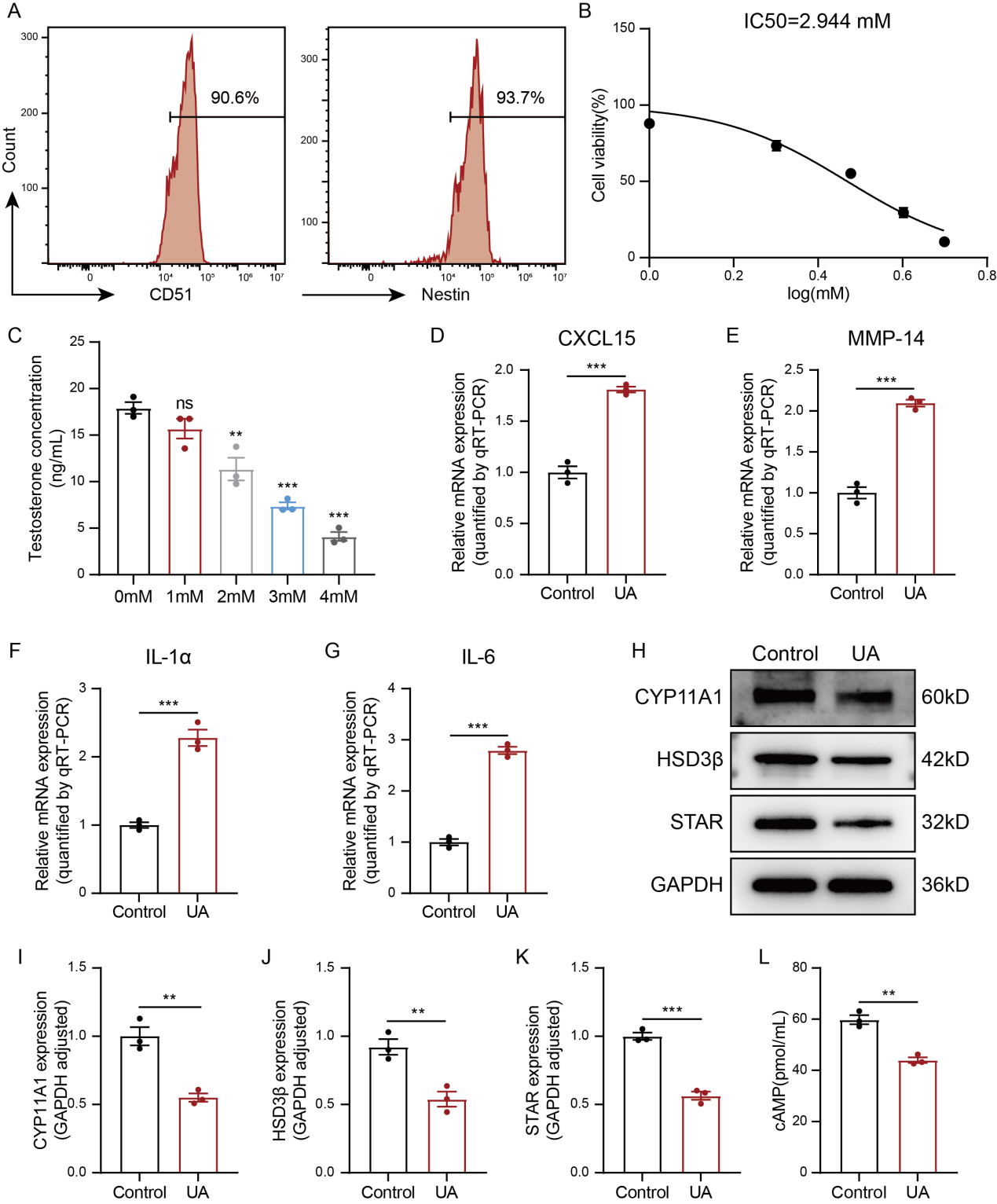


**Figure S3 | High uric acid environment drives SLCs to express SASP factor and reduces steroid synthesis.**

(A) Mixed cells were obtained by testicular digestion and filtration, and after 3 generations of cultivation in SLC proliferation medium, the expression of CD51 and Nestin was detected by flow cytometry.

(B) CCK8 assay was used to detect and analyze the *IC50* of uric acid in SLCs.

(C) Detecting testosterone concentration in culture medium under different concentrations of UA culture environment.

(D-G) qPCR analysis of relative mRNA expression of the SASP genes in SLCs of Control and UA groups.

(H-K) Western Blot analysis and quantification of LC markers expression after SLCs induced differentiation of Control and UA groups.

(L) ELISA experiment for detecting cAMP levels after SLCs induced differentiation of Control, UA.

Data are represented as mean ± SEM. In all bar graphs, each dot represents one biological replicate. *p < 0.05, **p < 0.01, ***p < 0.001; ns, no significance by unpaired Student’s t test (D-G and I-L) and one-way ANOVA (C).


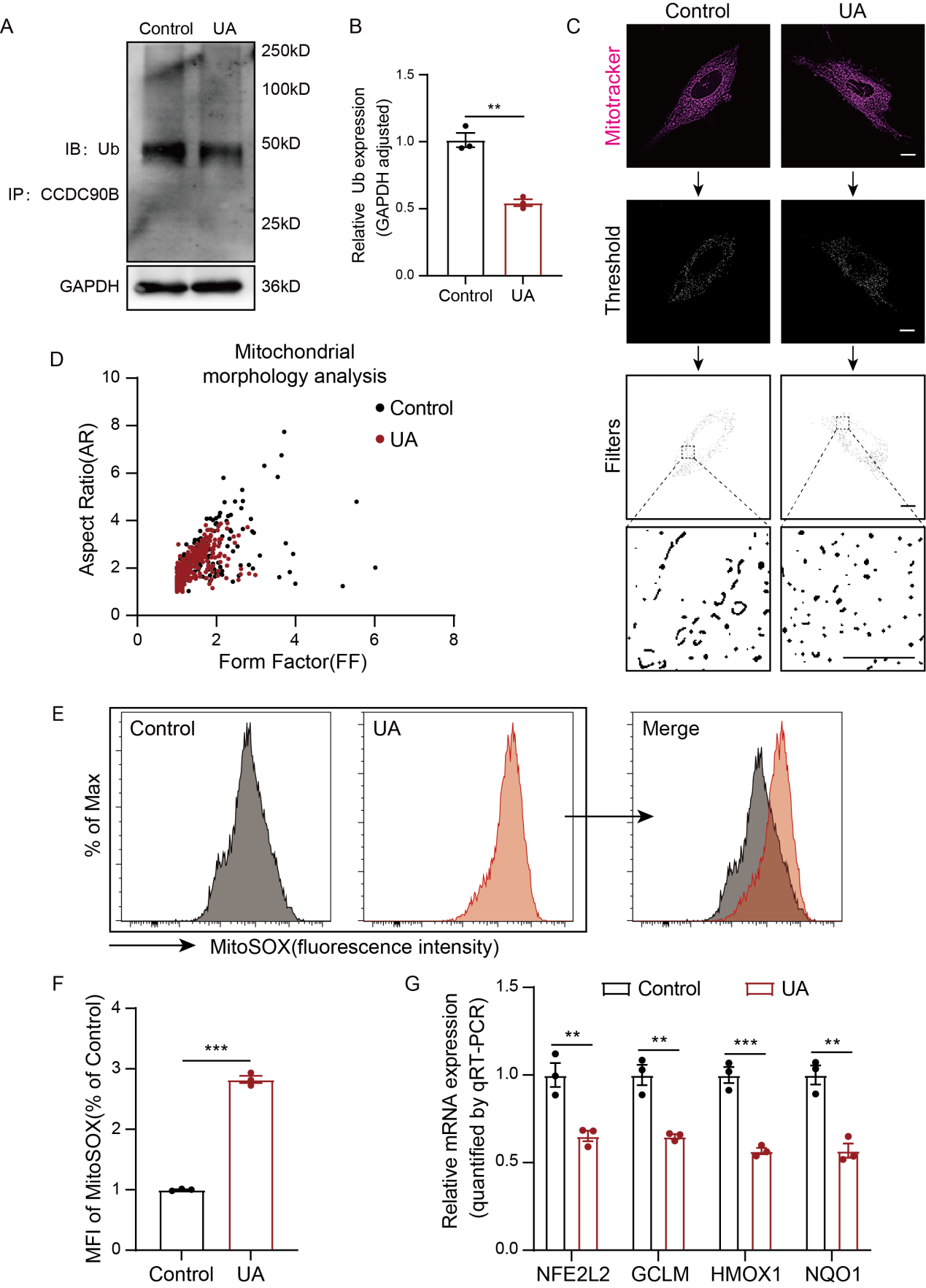


**Figure S4 | SLCs exhibit mitochondrial quality control imbalance under high uric acid conditions.**

(A and B) Western Blot analysis and quantification of the ubiquitination level of CCDC90B in SLCs of Control and UA groups.

(C) Representative immunostaining pictures of mitochondrial morphology of SLCs after treated with uric acid for 24 h. Mitochondria were marked by Mitotracker. Scale bar, 10 μm for original pictures and 5 μm for enlarged pictures.

(D) A plot of Aspect Ratio (AR) against Form Factor (FF) shows that particles in (C).

(E) Flow cytometry of mitochondrial ROS level stained with MitoSOX of SLCs of Control and UA groups.

(F) Quantification of mean fluorescence intensity of MitoSox in (E).

(G) qPCR analysis of relative mRNA expression of anti-oxidative genes of SLCs of Control and UA groups.

Data are represented as mean ± SEM. In all bar graphs, each dot represents one biological replicate. *p < 0.05, **p < 0.01, ***p < 0.001; ns, no significance by unpaired Student’s t test (B, F and G).


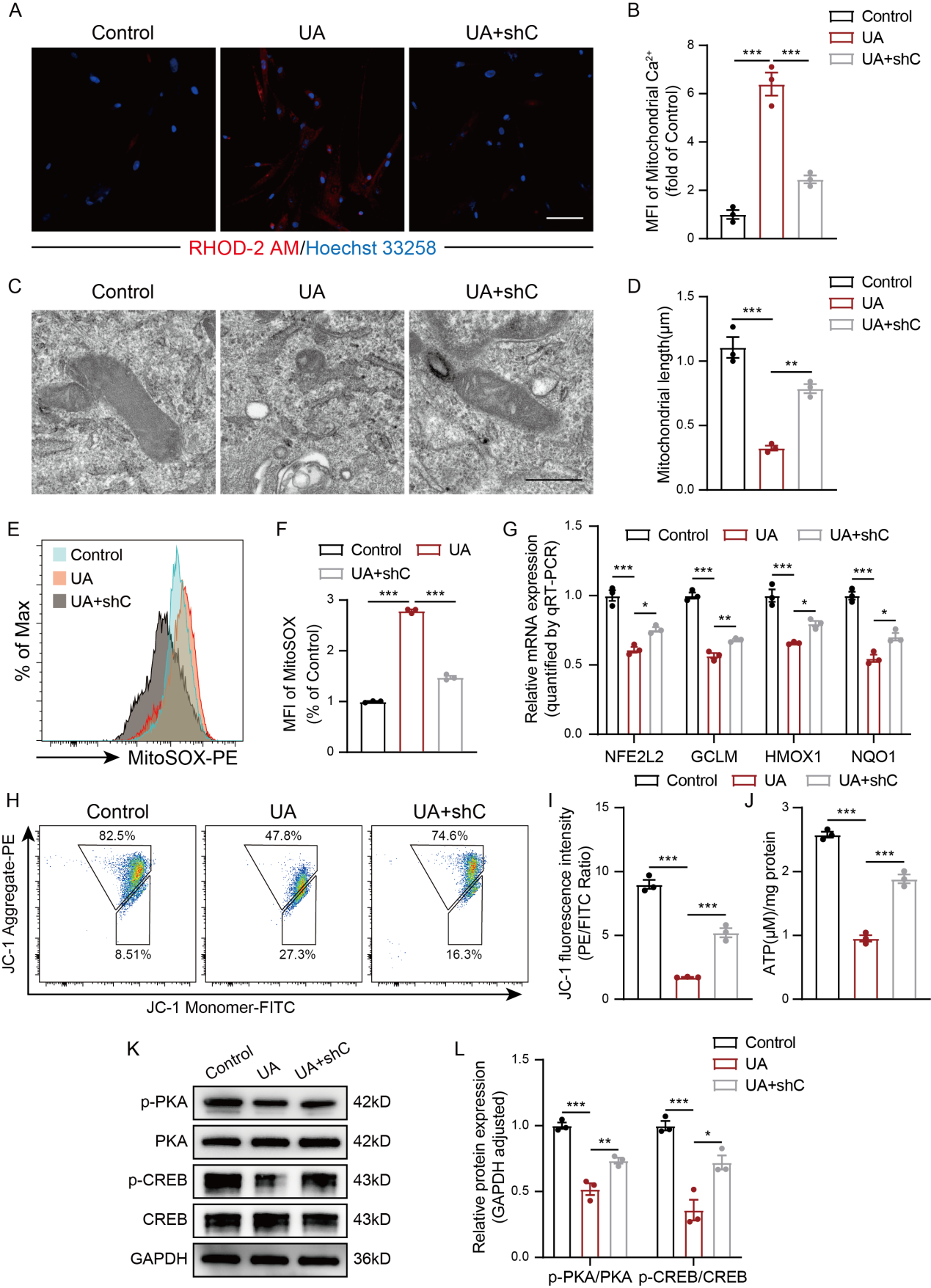


**Figure S5 | Interference with CCDC90B maintains mitochondrial quality control of SLCs under high uric acid conditions.**

(A) Representative immunofluorescence images of SLC mitochondrial calcium ion (RHOD-2 AM, red+) of Control, UA and UA + shC groups. (bar = 100 μm).

(B) Quantification of mean fluorescence intensity of mitochondrial calcium ion in (A).

(C) Representative transmission electron microscope images of the morphology of mitochondria in SLCs of Control, UA and UA + shC groups. (bar = 500 nm).

(D) Quantitative analysis of average value of mitochondrial length (μm) in (C).

(E) Flow cytometry of mitochondrial ROS level stained with MitoSOX of SLCs of Control, UA and UA + shC groups.

(F) Quantification of mean fluorescence intensity of MitoSox in (E).

(G) qPCR analysis of relative mRNA expression of anti-oxidative genes of SLCs of Control, UA and UA + shC groups.

(H) Flow cytometry analysis of mitochondrial membrane potential (MMP) probed with JC-1 in SLCs after treated with uric acid for 24 h.

(I) Quantitative analysis of the ratio of JC-1 aggregates (referred to high MMP, PE channel) /JC-1 monomers (referred to low MMP, FITC channel) in (H).

(J) Measurement of the intracellular ATP level in SLCs of Control, UA and UA + shC groups.

(K and L) Western Blot analysis and quantification of activation level of cAMP/PKA pathway after SLCs induced differentiation of Control, UA and UA + shC groups.

Data are represented as mean ± SEM. In all bar graphs, each dot represents one biological replicate. *p < 0.05, **p < 0.01, ***p < 0.001; ns, no significance by one-way ANOVA (B, D, F, G, I, J and L).


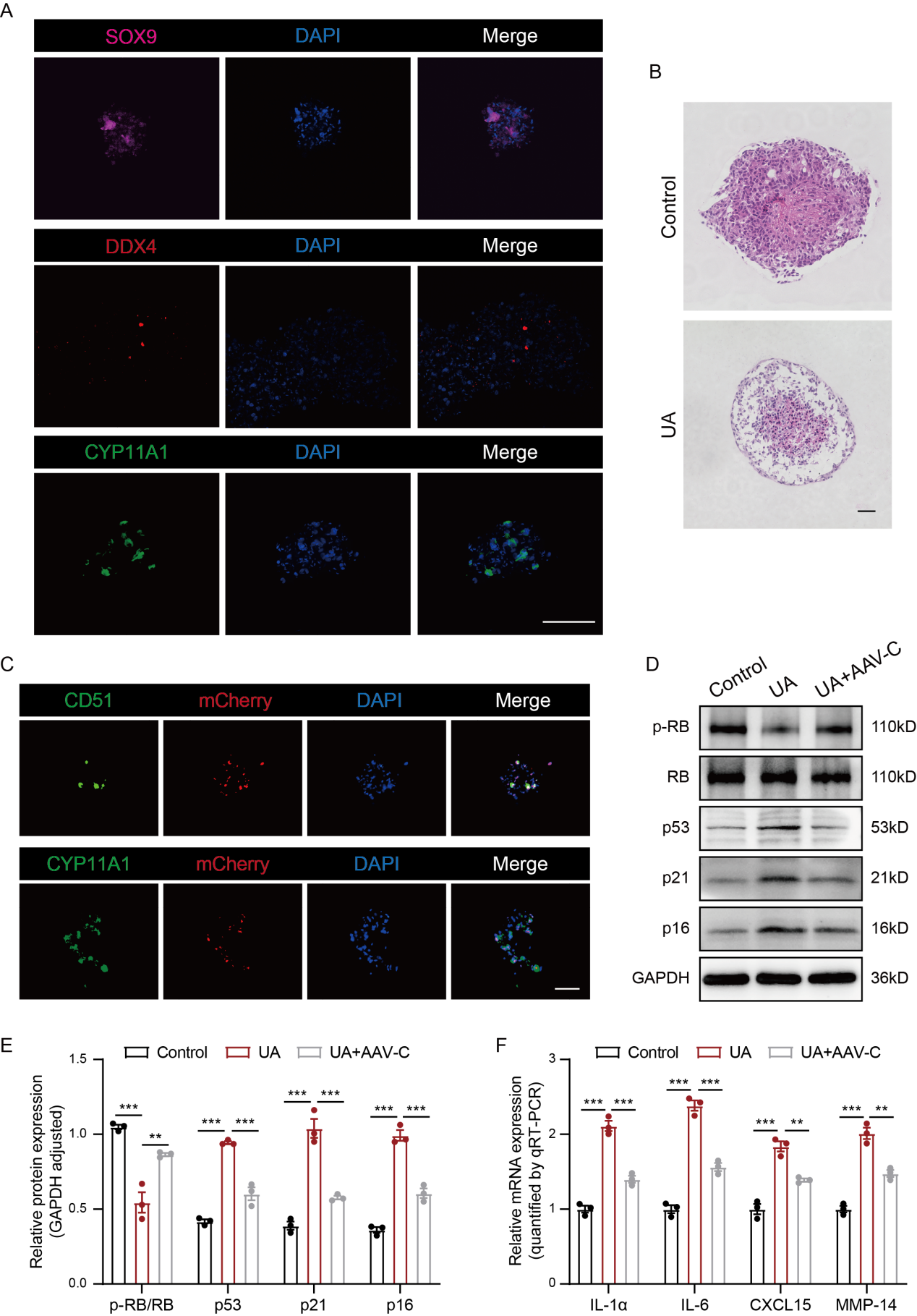


**Figure S6 | AAV-mediated downregulation of CCDC90B reduces the expression of aging-related genes in testicular organoids under high uric acid environment.**

(A) Immunofluorescent staining of testicular organoids depicting the localization of the Sertoli cell marker SOX9 (magenta staining), the germ cell marker DDX4 (red staining) and the Leydig cell marker CYP11A1 (green staining). (bar = 100 μm).

(B) Representative H&E staining pictures of testicular organoids from Control and UA groups. (bar = 10 μm).

(C) The testicular organoids were collected and immunofluorescent stained with SLC markers (CD51) and LC markers (CYP11A1) at 7 days after AAV injection. (bar = 50 μm).

(D and E) Western Blot analysis and quantification of senescence markers expression in testicular organoids of Control, UA and UA + AAV-C groups.

(F) qPCR analysis of relative mRNA expression of the SASP genes in testicular organoids of Control, UA and UA + AAV-C groups.

Data are represented as mean ± SEM. In all bar graphs, each dot represents one biological replicate. *p < 0.05, **p < 0.01, ***p < 0.001; ns, no significance by one-way ANOVA (E and F).


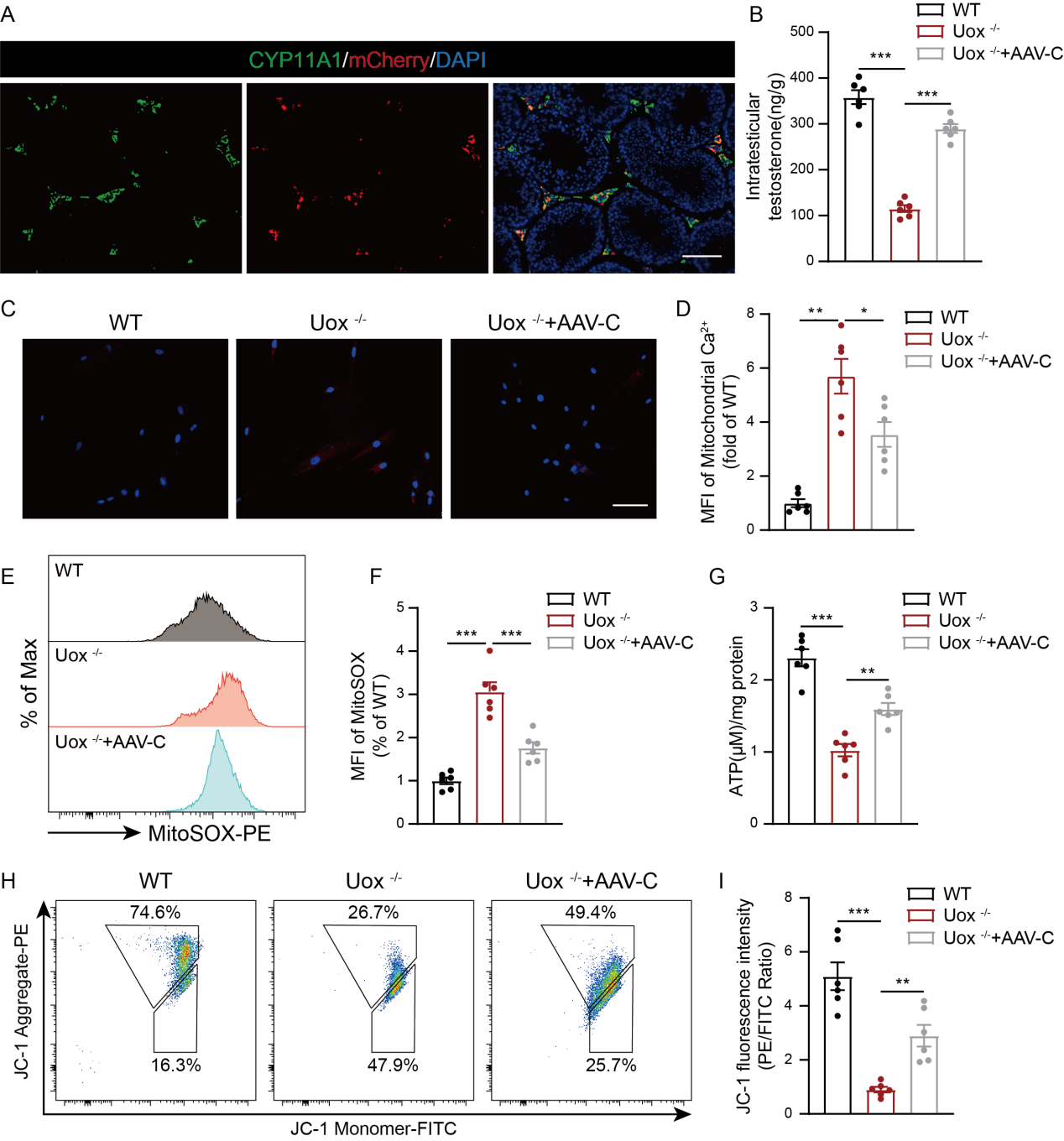


**Figure S7 | Interference with CCDC90B maintains mitochondrial quality control of SLCs in the hyperuricemia mice.**

(A) Representative immunofluorescence images of the testicular sections of WT mice injected with AAV8-CAG-mCherry. The testis tissues were collected and immunostained with LC markers (CYP11A1) at 7 days after AAV injection. (bar = 100 μm).

(B) Measurement of testosterone concentration in the testis.

(C) Representative immunofluorescence images of SLC mitochondrial calcium ion (RHOD-2 AM, red+) of WT, Uox^⁻/⁻^, Uox^⁻/⁻^ + AAV-C groups. (bar = 100 μm).

(D) Quantification of mean fluorescence intensity of mitochondrial calcium ion in (C).

(E) Flow cytometry of mitochondrial ROS level stained with MitoSOX of SLCs of WT, Uox^⁻/⁻^, Uox^⁻/⁻^ + AAV-C groups.

(F) Quantification of mean fluorescence intensity of MitoSox in (E).

(G) Measurement of the intracellular ATP level in SLCs of WT, Uox^⁻/⁻^, Uox^⁻/⁻^ + AAV-C groups.

(H) Flow cytometry analysis of mitochondrial membrane potential (MMP) probed with JC-1 in SLCs of WT, Uox^⁻/⁻^, Uox^⁻/⁻^ + AAV-C groups.

(I) Quantitative analysis of the ratio of JC-1 aggregates (referred to high MMP, PE channel) /JC-1 monomers (referred to low MMP, FITC channel) in (H).

Data are represented as mean ± SEM. In all bar graphs, each dot represents one biological replicate. *p < 0.05, **p < 0.01, ***p < 0.001; ns, no significance by one-way ANOVA (B, D, F, G and I).

**Supplementary Table 1 | Antibodies, Chemical reagents and Kits**

| **Antibodies** | **Company** | **Cat. No** |
| --- | --- | --- |
| CYP11A1 | GeneTex | GTX56293 |
| HSD3β | Santa Cruz | sc-515120 |
| STAR | proteintech | 67130-1-Ig |
| SOX9 | GeneTex | GTX01545 |
| DDX4 | GeneTex | GTX116575 |
| LHR | Abcam | ab204950 |
| CD51 | Santa Cruz | sc-13588 |
| p16 | Santa Cruz | sc-1661 |
| p21 | Santa Cruz | sc-136020 |
| p53 | Abcam | ab26 |
| RB | CST | 9313T |
| p-RB | CST | 8516T |
| CCDC90B | proteintech | 27126-1-AP |
| TOM20 | Santa Cruz | sc-136211 |
| LAMP1 | Santa Cruz | sc-20011 |
| Ubiquitin (Ub) | CST | 3936S |
| PKA | CST | 4782S |
| p-PKA | CST | 4781S |
| CREB | CST | 9197S |
| p-CREB | CST | 9198S |
| Nestin | Millipore | MAB353 |
| GAPDH | proteintech | 60004-1-Ig |
| Goat Anti-Rabbit IgG H&L (HRP) | Abcam | ab205718 |
| Goat Anti-Mouse IgG H&L (HRP) | Abcam | ab205719 |
| Goat anti-mouse IgG conjugated to Alexa Fluor™ 488 | Invitrogen | A-11001 |
| Goat anti-mouse IgG conjugated to Alexa Fluor™ 555 | Invitrogen | A-21422 |
| Goat anti-mouse IgG conjugated to Alexa Fluor™ 647 | Invitrogen | A-21235 |
| Goat anti-rabbit IgG conjugated to Alexa Fluor™ 488 | Invitrogen | A-11008 |
| Goat anti-rabbit IgG conjugated to Alexa Fluor™ 555 | Invitrogen | A-21428 |
| Goat anti-rabbit IgG conjugated to Alexa Fluor™ 647 | Invitrogen | A-21244 |
| **Chemical reagents** |  |  |
| Collagenase type IV | Gibco | 17104-019 |
| Dexamethasone | Sigma-Aldrich | D1756 |
| LIF | Millipore | LIF1010 |
| Insulin-transferrin-sodium selenite | Sigma-Aldrich | 11074547001 |
| Chick Embryo Extract | US Biologicals | C3999 |
| Non-Essential Amino Acids | HyClone | SH30050.03 |
| β-mercaptoethanol | Invitrogen | 21985023 |
| N2 supplement | Invitrogen | 17502001 |
| B27 supplement | Invitrogen | A1486701 |
| EGF | PeproTech | AF-100-15 |
| bFGF | Invitrogen | 13256029 |
| PDGF-BB | PeproTech | 100-14B |
| Oncostatin M | PeproTech | 300-10T |
| PDGF-AA | PeproTech | 100-13A |
| LH | R&D Systems | 8899-LH-010 |
| Forskolin, Fsk | Sigma-Aldrich | F6886 |
| Smoothened Agonist HCl (SAG) | Millipore | 566660 |
| Insulin-like growth factor 1 (IGF1) | PeproTech | 350-10 |
| Hoechst 33258 | Solarbio | C0021 |
| DAPI Solution | Sigma | D9542-1mg |
| TRIzol | Thermo | 15596018 |
| PMSF Solution (100mM) | Beyotime | ST507-10ml |
| Mitotracker Deep Red | Invitrogen | M22426 |
| MitoSOX™ Red | Invitrogen | M36007 |
| CellROX™ Green | Invitrogen | C10444 |
| Dihydroethidium | Sigma | 309800 |
| Matrigel | CORNING | 356237 |
| Gel digestion enzymes | Accurate Biotechnology | GXDLFA |
| KnockOut serum replacement (KSR) | Gibco | 10828-028 |
| **Kits** |  |  |
| Mouse Testosterone ELISA Kit | Fine Biotech | 40203ES80 |
| Mouse IL-6 ELISA Kit | Abclonal | RK00008 |
| Mouse IL-1 beta Fast ELISA Kit | Abclonal | RK04599 |
| Mouse TNF-alpha High Sensitivity ELISA Kit | Abclonal | RK04875 |
| Mouse Cathelicidin antimicrobial peptide (CAMP) ELISA Kit | Abclonal | RK02659 |
| Senescence β-Galactosidase Staining Kit | Beyotime | C0602 |
| Mitochondrial Calcium Assay Kit with Rhod-2 AM | Beyotime | S1062S |
| Cell Counting Kit (CCK-8) | Yeasen | 40203ES80 |
| Mitochondrial membrane potential assay kit with JC-1 | Beyotime | C2006 |
| CF640 Tunel Cell Apoptosis Detection Kit | Servicebio | G1505-100T |
| ATP Assay Kit | Beyotime | S0026 |
| BCA Protein Assay Kit | Beyotime | P0012S |
| HiFiScript All-in-one RT Master Mix Kit | cwbiotech | CW3371 |

**Supplementary Table 2 | Primers used for real-time PCR**

| mH3D3β-Forward | GCCTTCGAGACCCCAAGAAG |
| --- | --- |
| mH3D3β-Reverse | AAGGCTCCAGCTGGCATTAG |
| mCYP11A1-Forward | CACTGAGACTCCACCCCATC |
| mCYP11A1-Reverse | GGCAAAGCTAGCCACCTGTA |
| mHSD17β3-Forward | CAATGTTGTACTTATTAGTCGG |
| mHSD17β3-Reverse | ATTTCTAAGCCTTCAAGGTGT |
| mCYP17A1-Forward | GGAGAGTTTGCCATCCCGAA |
| mCYP17A1-Reverse | GAGCCTCTCCAATGCACGAT |
| mSTAR-Forward | CCGGAGCAGAGTGGTGTCA |
| mSTAR-Reverse | CAGTGGATGAAGCACCATGC |
| mNFE2L2-Forward | GGACATGGAGCAAGTTTGGC |
| mNFE2L2-Reverse | CCAGCGAGGAGATCGATGAG |
| mGCLM-Forward | AGTTGACATGGCATGCTCCG |
| mGCLM-Reverse | TCCATCTTCAATCGGAGGCG |
| mHMOX1-Forward | GAATCGAGCAGAACCAGCCT |
| mHMOX1-Reverse | CTCAGCATTCTCGGCTTGGA |
| mNQO1-Forward | CGCCTGAGCCCAGATATTGT |
| mNQO1-Reverse | GCACTCTCTCAAACCAGCCT |
| mIL-1α-Forward | ACGTCAAGCAACGGGAAGAT |
| mIL-1α-Reverse | AAGGTGCTGATCTGGGTTGG |
| mIL-6-Forward | CTCATTCTGCTCTGGAGCCC |
| mIL-6-Reverse | CAACTGGATGGAAGTCTCTTGC |
| mCXCL15-Forward | CTAGGCATCTTCGTCCGTCC |
| mCXCL15-Reverse | TGGGACTGCTATCACTTCCTT |
| mMMP-14-Forward | ATGGCCCCCTTTTACCAGTG |
| mMMP-14-Reverse | GTGACCCTGACTTGCTTCCATA |
| m18S-Forward | GTAACCCGTTGAACCCCATT |
| m18S-Reverse | CCATCCAATCGGTAGTAGCG |
